# A chromosome-scale reference genome of *Aquilegia oxysepala* var. *kansuensis*

**DOI:** 10.1038/s41438-020-0328-y

**Published:** 2020-07-01

**Authors:** Jinghe Xie, Haifeng Zhao, Kunpeng Li, Rui Zhang, Yongchao Jiang, Meimei Wang, Xuelian Guo, Ben Yu, Hongzhi Kong, Yuannian Jiao, Guixia Xu

**Affiliations:** 1 State Key Laboratory of Systematic and Evolutionary Botany, CAS Center for Excellence in Molecular Plant Sciences, Institute of Botany, Chinese Academy of Sciences, Beijing, 100093 China; 2 University of Chinese Academy of Sciences, Beijing, 100049 China

**Keywords:** Plant evolution, Comparative genomics

## Abstract

The genus *Aquilegia* (Ranunculaceae) has been cultivated as ornamental and medicinal plants for centuries. With petal spurs of strikingly diverse size and shape, *Aquilegia* has also been recognized as an excellent system for evolutionary studies. Pollinator‐mediated selection for longer spurs is believed to have shaped the evolution of this genus, especially the North American taxa. Recently, however, an opposite evolutionary trend was reported in an Asian lineage, where multiple origins of mini- or even nonspurred morphs have occurred. Interesting as it is, the lack of genomic resources has limited our ability to decipher the molecular and evolutionary mechanisms underlying spur reduction in this special lineage. Using long-read sequencing (PacBio Sequel), in combination with optical maps (BioNano DLS) and Hi–C, we assembled a high-quality reference genome of *A. oxysepala* var. *kansuensis*, a sister species to the nonspurred taxon. The final assembly is approximately 293.2 Mb, 94.6% (277.4 Mb) of which has been anchored to 7 pseudochromosomes. A total of 25,571 protein-coding genes were predicted, with 97.2% being functionally annotated. When comparing this genome with that of *A*. *coerulea*, we detected a large rearrangement between Chr1 and Chr4, which might have caused the Chr4 of *A. oxysepala* var. *kansuensis* to partly deviate from the “decaying” path that was taken before the split of *Aquilegia* and *Semiaquilegia*. This high-quality reference genome is fundamental to further investigations on the development and evolution of petal spurs and provides a strong foundation for the breeding of new horticultural *Aquilegia* cultivars.

## Introduction

The genus *Aquilegia* (Ranunculaceae), commonly known as columbine, consists of ~70 species that are widely distributed in the temperate zones of the Northern Hemisphere^1^. Numerous species and varieties of this genus have been cultivated as garden ornamentals for centuries due to their attractive flowers with unusual characteristics, including petaloid sepals and petal spurs of diverse shape and size^2–4^. Particularly, the length of spurs (∼1–16 cm) varies dramatically, matching the tongue length of the corresponding pollinators; this has made the genus a model system for research on pollinator-driven diversification^2,5^. In fact, the multiple origins of species with longer petal spurs from those with shorter ones in the North American *Aquilegia* clade have become textbook examples of pollinator shift-mediated adaptive evolution^5–7^. Recently, however, one Asian *Aquilegia* lineage was revealed to have experienced multiple origins of mini- or even nonspurred morphs, indicating an opposite trend of spur evolution^8,9^. The genus, therefore, is also an excellent system for the study of spur reduction. Deciphering the developmental and evolutionary mechanisms underlying these morphological changes would not only facilitate our understanding of species diversification but also provide a good foundation for the breeding of promising horticultural *Aquilegia* cultivars.

Genomic resources are essential and attainable for developmental and evolutionary studies. To date, only one genome in the *Aquilegia* genus has been sequenced (*A*. *coerulea* reference genome v3.1)^10^. *A*. *coerulea* has flowers with long petal spurs and belongs to the North American clade^10,11^; its genomic resources have facilitated numerous studies concerning spur elongation, adaptation, and speciation^12–18^. However, the application of these resources to address the problems of spur reduction is limited because *A*. *coerulea* diverged from the Asian lineage approximately 4.8 million years ago (Mya)^11^. Moreover, several lines of evidence suggest a “decaying” nature of *Aquilegia* Chr4, which has likely been evolving under reduced purifying and/or background selection and has a strikingly higher level of polymorphism than the rest of the genome^10^. This further aggravates the difficulty in referring to the genome of a phylogenetically far-related species.

**Fig. 1:**
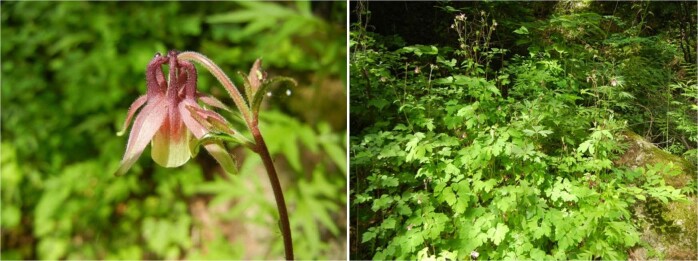
Images of *A. oxysepala* var. *kansuensis*

Here, we report the genome of *A. oxysepala* var. *kansuensis* (Fig. 1), a species that is sister to the nonspurred taxon in Asia^8^. We assembled a chromosome-scale reference genome by long-read sequencing (PacBio Sequel system), optical map (BioNano DLS) scaffolding, and further anchoring the scaffolds to pseudochromosomes using Hi–C. Comparison of the *A. coerulea* and *A. oxysepala* var. *kansuensis* genomes revealed a large chromosomal rearrangement between Chr1 and Chr4, and we cataloged the species-specific genes. Notably, we found that, unlike Chr4 in other *Aquilegia* taxa, this chromosome in *A. oxysepala* var. *kansuensis* is not evolving entirely under reduced purifying selection due to the rearrangement event.

## Results

### Genome size and heterozygosity estimation

We used a single individual of *A. oxysepala* var. *kansuensis* that was collected from Yuzhong County, Gansu Province, China, for whole-genome sequencing. We noticed that a new name, *A*. *yangii*, has recently been given to plants of this and many other populations^19^. However, we decided to stay with the original name because the new one is still poorly known. To guide genome sequencing and assembly, we estimated the genome size of *A. oxysepala* var. *kansuensis* using flow cytometry^20^ and K-mer analysis. Briefly, flow cytometry indicated that *A. oxysepala* var. *kansuensis* had a genome size of 312 Mb (Fig. S1). For K-mer analysis, we obtained 18.3 Gb short paired-end reads by Illumina sequencing; 18.2 Gb were retained after the removal of low-quality reads (Table S1). Based on the total number of 17-mers and the depth of the main peak in the 17-mer frequency distribution (Fig. S2), we estimated that the genome size and heterozygosity rate of *A. oxysepala* var. *kansuensis* were 349 Mb and 0.15%, respectively (Table S2). To ensure we had enough data for genome assembly, we decided to use 349 Mb as a reference for further sequencing.

### Sequencing and assembly of the genome

**Fig. 2:**
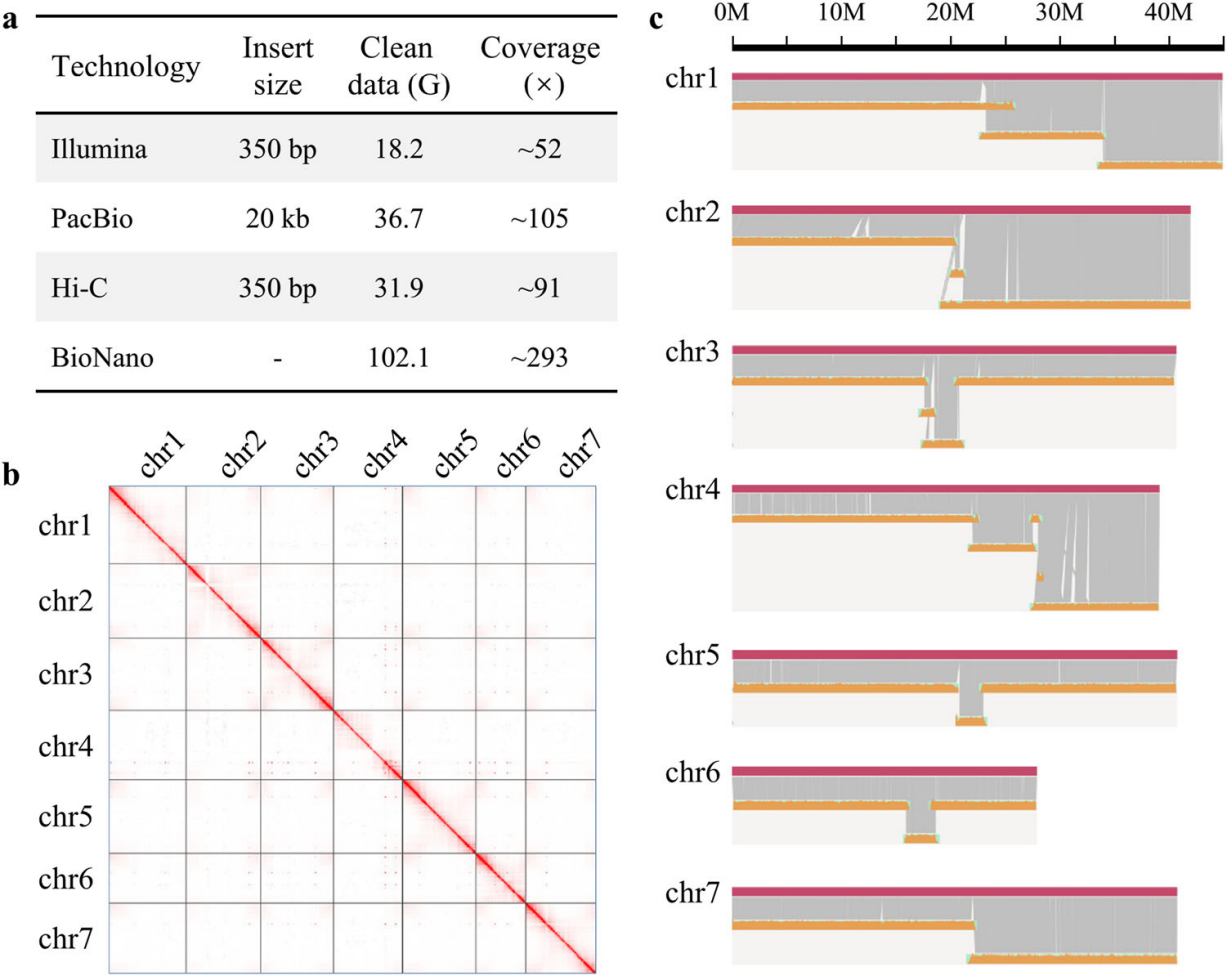
Genome assembly of *A. oxysepala* var. *kansuensis*. **a** Summary of sequencing data used for genome assembly. Sequencing coverage was calculated based on the genome size obtained from K-mer analysis (349 Mb). For BioNano, effective coverage of the reference is shown. **b** Hi–C heatmap showing interactions among the 7 pseudochromosomes. **c** Alignment between the BioNano DLS maps and the final genome assembly

To obtain a high-quality genome, three technologies were applied: PacBio SMRT sequencing (36.7 Gb, ~105×; Table S3, Fig. 2a), BioNano DLS optical mapping (102.1 Gb, ~293×; Table S4, Fig. 2a) and Hi-C mapping (31.9 Gb, ~91×; Table S5, Fig. 2a). The PacBio long-read assemblies showed high contiguity and resulted in a total of only 852 contigs with an N50 of 2.2 Mb, and the longest one was approximately 7.8 Mb (Table S6). After adding the optical mapping data from BioNano DLS, we were able to assemble the contigs into scaffolds. A total of 663 scaffolds (total length = 297.8 Mb) were produced, among which 21 were hybrid scaffolds (total length = 284.8 Mb) encompassing, in most cases, entire chromosome arms. The longest scaffold reached 41.4 Mb, and the N50 was 40.9 Mb. We then anchored all these scaffolds to 7 pseudochromosomes using the Hi–C data in 3D de novo assembly (3D-DNA) software^21^. Eventually, a high-quality chromosome-level *A. oxysepala* var*. kansuensis* assembly (679 scaffolds) was obtained, with 7 pseudochromosomes accounting for 94.6% (277.4/293.2) of the total genome length (Table 1).

**Table 1:** Assembly and annotation statistics of the *A*. *oxysepala* var. *kansuensis* and *A*. *coerulea* genomes

	*A. oxysepala* var. *kansuensis*	*A. coerulea*
Technology	Illumina/PacBio/BioNano/Hi-C	Sanger/Illumina
Number of contigs	852	7930
Contig N50 (Mb)	2.22	0.11
Number of scaffolds	679	1034
Scaffold N50 (Mb)	40.90	43.63
Number of pseudochromosomes	7	7
Length of pseudochromosomes (Mb)	277.44	295.11
Total length (Mb)	293.21	306.52
Gap (%)	0.93	4.82
Number of protein-coding genes	25,571	30,023
BUSCO	C: 93.2% F: 1.9% M: 4.9%	C: 93.0% F: 2.1% M: 4.9%

Five approaches were utilized to evaluate the quality of the assembly. First, the Illumina short reads were mapped back to the assembled contigs using Burrows–Wheeler Aligner (BWA) software^22^. The mapping rate of paired-end reads reached 97.2%, indicating high completeness and accuracy of the final assembly. Second, to assess the completeness of the assembly, we performed benchmarking universal single-copy ortholog (BUSCO)^23^ analysis by searching against the 1440 conserved single-copy genes in plants and identified 1342 (93.2%) complete BUSCOs (Table 1). Third, when the BioNano assembly consensus genome maps (CMAPs) were aligned to the 7 in silico maps of the *A. oxysepala* var*. kansuensis*, a total of 277.3 Mb (unique aligned length) were covered, validating 94.6% of the assembly (Table S7). Fourth, we extracted single nucleotide polymorphisms (SNPs) of the whole-genome using SAMtools^24^ and found that the proportions of heterozygous and homozygous SNPs were 0.022% and 0.001%, respectively, suggesting high accuracy of the assembly. Finally, we used the LTR assembly index (LAI)—a standard for evaluating the assembly of repeat sequences—to assess assembly continuity^25^. We found that the LAI score of the *A*. *oxysepala* var. *kansuensis* assembly reached 16.7, which was much higher than that of *A*. *coerulea* (LAI = 12.6). Both genomes could be classified as reference quality, similar to the quality of the *Arabidopsis* TAIR10 genome (LAI = 14.9)^25^. Taken together, these results suggest that the genome assembly had very high continuity, completeness, and correctness.

### Annotation of the genome

**Fig. 3:**
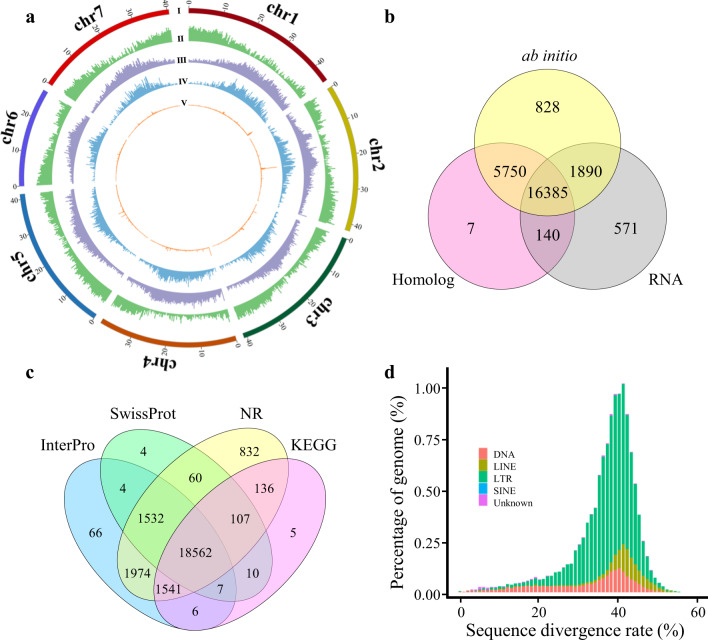
Annotation of the *A. oxysepala* var. *kansuensis* genome. **a** Distribution of genes and different types of repeat elements: **I**, Legends of seven pseudochromosomes; **II**, gene density; **III**, transposon density; **IV**, transposable element protein density; and **V**, tandem repeat density. **b** Number of genes predicted by different approaches: ab initio (yellow), homology-based (pink) and transcriptome-assisted (gray). **c** Number of functionally annotated genes based on various databases. **d** Accumulation history of different kinds of repeat elements

Two methods (i.e., homology alignment and de novo annotation) were used to identify repeats in the assembly. Among the five major types of repeats detected (DNA transposon, LINE, LTR, SINE, and unknown), LTRs comprised the largest proportion (35.88%, total length 105.2 Mb). Unknown repeats ranked second, occupying 6.46% (total length 18.9 Mb) of the genome. DNA transposons, LINEs, and SINEs accounted for 2.27%, 1.06%, and 0.01% of the genome, respectively (Fig. 3a, d). Thus, altogether, 45.68% of the genome was predicted to be repeats.

A combination of three methods, including homology-based prediction, ab initio prediction and transcriptome-assisted prediction, was employed to identify protein-coding genes. Sequences from six species were used as references (see Section “Materials and methods”) for homology-based prediction, which generated a total of 22,282 gene models (Fig. 3b). For ab initio prediction, a list of 24,853 putative gene models (Fig. 3b) was obtained by integrating the results from several software packages (see Section “Materials and methods”). To enhance transcriptome-assisted prediction, we performed Iso-Seq and RNA-Seq on various plant tissue samples of different developmental stages (see Section “Materials and methods”). Specifically, Iso-Seq was conducted on five different samples, yielding 46.7 Gb clean data; pooled (equal-amount) total RNA from these samples was also subjected to RNA-Seq, producing 12.8 Gb clean data (Table S8). By applying these data, 18,986 genes were identified (Fig. 3b). The three datasets were then merged together to generate a nonredundant reference gene set containing 25,571 protein-coding genes, with 24,913 on the 7 chromosomes and 658 on the unassigned scaffolds (Fig. 3b). These genes had transcripts that were on average 3,447 bp, including 5 exons with a mean length of 242 bp. We further detected that 24,744, 20,286, 23,692, and 20,374 genes showed significant similarity to known proteins in the NR, Swiss-Prot, InterPro, and KEGG databases, respectively (Fig. 3c). Integration of the four datasets led to the assignment of potential functions to 24,846 (97.2%) of the 25,571 protein-coding genes in the *A. oxysepala* var. *kansuensis* genome. We also identified 930 tRNAs, 483 rRNAs (5S, 5.8S, 18S, and 28S), 696 snRNAs, and 275 miRNAs.

### Comparison of the genomes of the two *Aquilegia* taxa

**Fig. 4:**
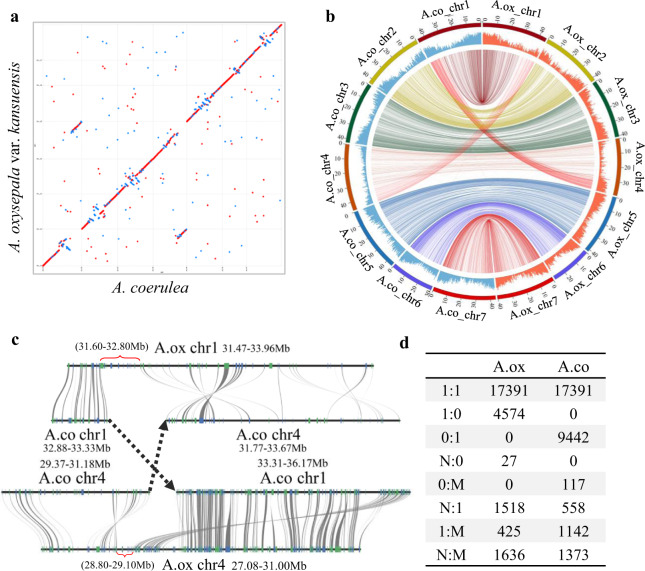
Genome comparison between *A. oxysepala* var. *kansuensis* and *A. coerulea*. **a** Dot-plot alignment between the two taxa. **b** Circos plot showing syntenic relationships and chromosomal rearrangements between the two genomes. **c** Synteny of genes located in and around the breakpoint regions of chromosomes 1 and 4. Red braces stand for the breakpoint regions in *A*. *oxysepala* var. *kansuensis*. **d** Comparison of gene numbers in different classes of orthologous groups

Using MUMmer software (v3.23), we aligned the *A. oxysepala* var. *kansuensis* genome assembly with the *A. coerulea* genome (v3.1)^10^. For convenience, the chromosomes of these two species will hereafter be referred to as A.ox_chr1–7 and A.co_chr1–7, respectively. The two genomes showed extensive synteny except for one large rearrangement between Chr1 and Chr4 (Fig. 4a, b). Specifically, relative to the *A. coerulea* genome, there was a reciprocal chromosomal translocation between A.ox_chr1 and A.ox_chr4. To confirm the correct assembly around the breakpoints of the translocated regions, we performed two different analyses. First, we mapped all PacBio long reads to our assembly to determine if there were misjoins caused by a shortage of read mapping evidence near the breaking area. The results showed that the mapping coverage spanning and flanking the area was similar to that in other regions (Fig. S3). Second, we rearranged the two concerned chromosomes, referring to the *A. coerulea* genome, and thereby created two hypothesized chromosomes (h_A.ox_chr1 and h_A.ox_chr4) that shared chromosome-wide synteny with A.co_chr1 and A.co_chr4, respectively. We then mapped the Hi–C data to h_A.ox_chr1 and h_A.ox_chr4 using Juicer software^26^. If the two hypothesized chromosomes represented the correct assembly, we would expect a smoother Hi–C interaction heatmap. On the contrary, we observed obvious chromogram discontinuities, indicating misjoins (Fig. S4b). Likewise, when aligning the Hi–C data to the genome of *A. coerulea*, we also found apparent conflicts on A.co_chr1 and A.co_chr4 (Fig. S4c). These results together suggest that the rearrangement between Chr1 and Chr4 is likely to be real. We further zoomed in the rearrangement region to examine whether there were special genes located around the breakpoints. Adjacent syntenic blocks were compared in detail to determine the exact break sites. As shown in Fig. 4c, the 32.88–33.33 Mb region on A.co_chr1 could be aligned to the 31.47–31.6 Mb of A.ox_chr1, while the downstream 33.31–36.17 Mb aligned to the 29.10–31.00 Mb region of A.ox_chr4. The 29.37–31.18 Mb region of A.co_chr4 was alignable with the 27.08–28.80 Mb region of A.ox_chr4, whereas the downstream 31.77–33.67 Mb shared synteny with the 32.80–33.96 Mb region of A.ox_chr1. From these results, we determined the breakpoint regions, including the 31.60–32.80 Mb region on A.ox_chr1 and the 28.80–29.10 Mb region on A.ox_chr4. These two fragments each contained 16 and 7 genes (Fig. 4c), among which 3 and 2, respectively, had no homologous counterparts in the *A. coerulea* genome.

Compared with the genome of *A. coerulea* (v3.1), in which 30,023 protein-coding genes were annotated^10^, the genome of *A. oxysepala* var. *kansuensis* contained 4452 fewer genes. To further understand the gene content differences between these two genomes, we first clustered their genes into different orthogroups using OrthoFinder software (v2.3.3)^27^. Among the 55,594 genes, 17,391 pairs were one-to-one orthologs. A total of 14,160 genes (4601 in *A. oxysepala* var. *kansuensis* and 9559 in *A. coerulea*) formed clusters (X:0 clusters, X ≥ 1) with genes from only one of the two species (Fig. 4d). Although in other gene clusters, copy number differences were found between these two species, the aggregate numbers (3579 vs. 3073) were similar (Fig. 4d). Thus, the possession of more X:0 clusters in *A. coerulea* was the main reason for the interspecies gene number discrepancy. We further conducted a new round of ortholog clustering to identify genes in these X:0 clusters that were likely to be species-specific by including sequences from *Amborella trichopoda*, *Oryza sativa*, *Vitis vinifera*, *Arabidopsis thaliana*, and *Papaver somniferum*. This analysis revealed that 2250 and 5119 genes were specific to *A. oxysepala* var. *kansuensis* and *A. coerulea*, respectively.

**Fig. 5:**
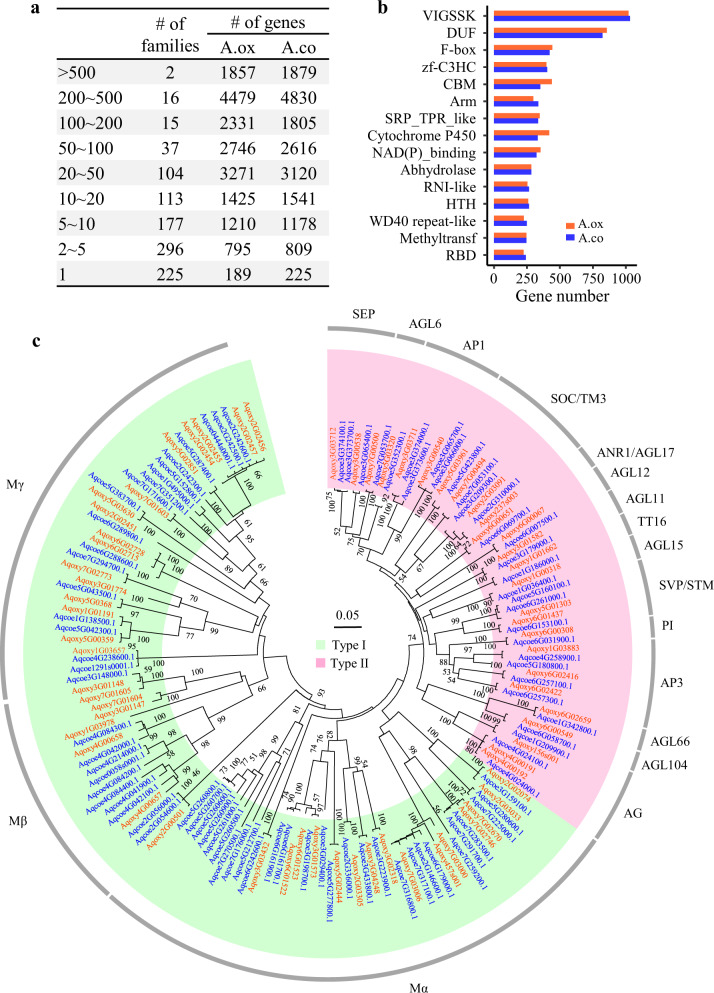
Gene family comparison between *A.oxysepala* var. *kansuensis* and *A. coerulea*. **a** The number of gene families in each size category and the number of genes from each species. The size of each gene family was defined by referring to the genes in *A*. *coerulea*. Note that gene family classifications are not mutually exclusive. **b** Comparison of gene numbers in the top 15 families (ranked by size). **c** Phylogenetic tree (neighbor joining, NJ) of MADS-box genes from the two species constructed with the *p*-distance model (1000 bootstrap replications)

Moreover, we classified genes into different families to further elucidate the expansion/contraction differences in certain categories between *A. oxysepala* var. *kansuensis* and *A. coerulea* (Fig. 5a). By referring to a database where 13,867 genes in *A. coerulea* were assigned to 985 families, we were able to put 13,860 genes from *A. oxysepala* var. *kansuensis* into 955 families. We found that the two species showed clear differences in the number of genes in some functionally important families (e.g., DUF, F-box, CBM, cytochrome P450, and MADS-box) (Fig. 5b, c and Table S9). For example, this detailed analysis identified 65 and 88 MADS-box genes from *A. oxysepala* var. *kansuensis* and *A. coerulea*, respectively. In line with the contrast evolutionary patterns of Type I (fast birth-and-death) and Type II (highly conserved) MADS-box genes, the two species contained comparable numbers of Type II genes, while *A. coerulea* contained 18 more Type I genes (Fig. 5c). Because Type II genes play important roles in flower development^28,29^, we further examined the copy number difference in each subfamily between the two species. In each of the *APETALA1* (*AP1*), *SEPALLATA* (*SEP*), *APETALA3* (*AP3*), and *SHORT VEGETATIVE PHASE* (*SVP*) subfamilies, *A. coerulea* contained one more copy. Genes in the first three subfamilies usually determine floral organ identity^28^, while those in the *SVP* subfamily control flowering time^28^. These differences may have impacted the difference in flower traits between *A. oxysepala* var. *kansuensis* and *A. coerulea*; further investigations on these genes would be of interest.

**Fig. 6:**
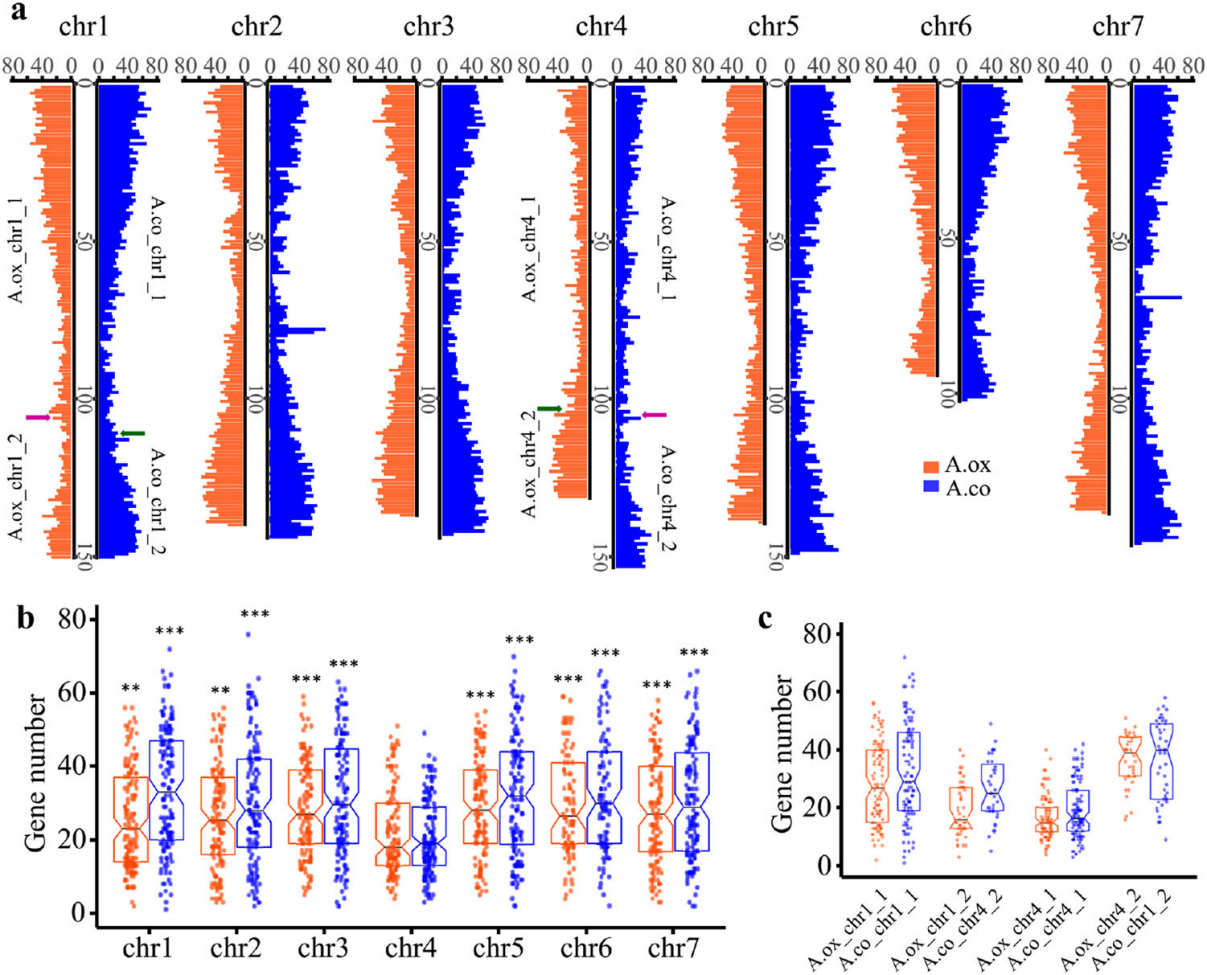
Distribution and significance test of gene density. **a** Distribution of gene number in each 300 kb window. The arrows on chromosomes 1 and 4 designate rearrangement breakpoints. Pink arrows show that A.ox_chr1_2 is homologous to A.co_chr4_2, while dark-green arrows show the homology of A.ox_chr4_2 with A.co_chr1_2. **b** Boxplot of gene density on each chromosome. Each point represents the number of genes in a 300 kb window. In the boxplots, each box indicates the lower quartile, median, and upper quartile values. Pairwise significance tests between Chr4 and the other chromosomes within each species were performed. Asterisks indicate significance level: **P < 0.01, ***P < 0.001. Detailed *P* value for each comparison is provided in Table S10. **c** Gene density on different homologous fragments. Detailed *P* value for each comparison is provided in Table S12

**Fig. 7:**
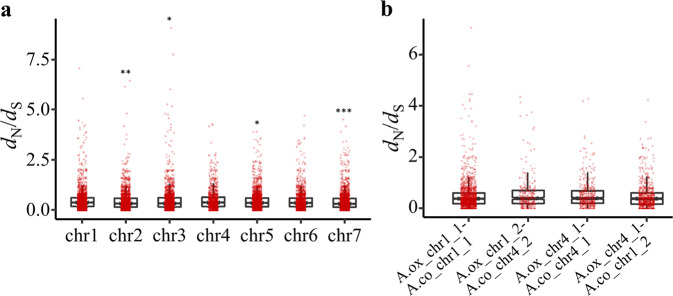
Distribution and significance test of *d*_N_/*d*_S_ values. **a** Boxplot of *d*_N_/*d*_S_ values calculated between one-to-one orthologs. Genes were assigned to different chromosomes according to the *A*. *oxysepala* var. *kansuensis* genome. Pairwise significance tests between Chr4 and the other chromosomes were performed. Asterisks indicate significance level: *P < 0.05, **P < 0.01, ***P < 0.001. Detailed *P* value for each comparison is provided in Table S11. **b** Boxplot of *d*_N_/*d*_S_ values for one-to-one orthologs distributed on the rearranged chromosome segments. Detailed *P* value for each comparison is provided in Table S13

Previous studies have suggested that the entire Chr4 in *Aquilegia* had a unique evolutionary pattern, with approximately twice the level of polymorphism of the rest of the genome, which might have been caused by reduced purifying selection^10^. For this reason, we performed two types of analyses. First, we calculated the density of genes on all seven chromosomes (Fig. 6a). We found that the density of genes on A.ox_chr4 was significantly lower than that on the other chromosomes (Benjamini–Hochberg adjusted *P* ≤ 0.01, one-sided Mann–Whitney *U* test; Fig. 6b and Table S10). We then calculated the *d*_N_/*d*_S_ ratio of all one-to-one orthologs between *A. oxysepala* var. *kansuensis* and *A. coerulea*. Regardless of the translocation, when comparing genes on A.ox_chr4 with those on the other chromosomes, we found that the *d*_N_/*d*_S_ ratios on this chromosome were the highest, although not every comparison was significant after multiple-testing correction (Fig. 7a and Table S11). This indicates that genes on this chromosome are likely to be evolving under fewer functional constraints.

However, because there was a rearrangement between A.ox_chr4 and A.ox_chr1, it was not clear whether the entire A.ox_chr4 or just the part that was homologous to A.co_chr4 had this evolutionary pattern. We thus separated the two focal chromosomes into four parts (i.e., A.ox_chr1_1, A.ox_chr1_2, A.ox_chr4_1, and A.ox_chr4_2) according to their homologous relationships with the *A. coerulea* genome and made further comparisons. We found that A.ox_chr4_1 and A.ox_chr1_2, which were homologous to Chr4 of *A. coerulea*, had the lowest gene density (Benjamini–Hochberg adjusted *P* < 0.001, one-sided Mann–Whitney *U* test; Fig. 6c, Table S12). When comparing *d*_N_/*d*_S_ values, not surprisingly, we found that the highest values were also from genes on A.ox_chr4_1 (median = 0.39) and A.ox_chr1_2 (median = 0.38) (Fig. 7b). However, statistical tests for comparison between A.ox_chr4_1 and other chromosomes/chromosomal segments were significant (Benjamini–Hochberg adjusted *P* < 0.03, one-sided Mann–Whitney *U* test; Table S13), while only two of the tests for A.ox_chr1_2 were significant (Benjamini–Hochberg adjusted *P* < 0.05, one-sided Mann–Whitney *U* test; Table S13). The discrepancy between these two segments indicates that they may have evolved under different constraints.

Previous studies suggested that *Aquilegia* species are ancient tetraploids, but the timing of the whole-genome duplication (WGD) event is debated^30,31^. Guo and colleagues (2018) held that this event occurred after the divergence of Papaveraceae and Ranunculaceae, while Akoz and Nordborg (2019) proposed that it was shared by all eudicots. To better understand this event, we identified all the within-genome syntenic regions of the *A*. *oxysepala* var. *kansuensis* genome and calculated the synonymous substitution rate (*d*_S_) of each pair of collinear paralogs using the modified Nei–Gojobori model. For comparison, the *d*_S_ values of collinear paralogs in *A*. *coerulea* and grape and those of the one-to-one orthologs between *A*. *oxysepala* var. *kansuensis* and grape were also estimated. The distributions of *d*_S_ values for collinear paralogs in the two *Aquilegia* species peaked at the same position (Fig. S7), confirming that *Aquilegia* species are ancient tetraploids. Although the grape paralogs had a similar peak value on the distribution plot of *d*_S_ values, the *d*_S_ distribution of orthologs of *A*. *oxysepala* var. *kansuensis* and grape peaked at a higher value, indicating that the WGD event occurred after the divergence of *Aquilegia* and grape. Therefore, our results strongly support that the most recent WGD in *Aquilegia* occurred after the divergence of Papaveraceae and Ranunculaceae, which is not consistent with the previously hypothesized pre-eudicot tetraploidization^30^.

## Discussion

### A high-quality genome of *A. oxysepala* var. *kansuensis*

In this study, by combining PacBio, BioNano, and Hi–C data, we have built a nearly complete assembly of the genome of *A. oxysepala* var. *kansuensis*. The evaluation results show that the genome is of high quality. In comparison with the *A*. *coerulea* genome v3.1, the assembly of *A. oxysepala* var*. kansuensis* has better contiguity, with longer contig N50, fewer contigs/scaffolds, and a lower percentage of gaps (Table 1). Notably, the 7 chromosomes of *A. oxysepala* var. *kansuensis* were supported by superscaffolds almost as long as chromosome arms. The biggest difference between these two genomes is the total number of protein-coding genes; the *A. coerulea* genome is annotated with 4452 more genes. Our detailed comparison revealed that a great part of this discrepancy can be attributed to the many more species-specific genes in *A. coerulea*. Two underlying reasons might account for this. First, these genes were generated de novo or were lost in other species, which may have played important roles in interspecific morphological or physiological divergence. Second, since gene predictions are not error-free, it is possible that some of them have been generated by misannotation. The observation that the mRNA length of the species-specific group was significantly shorter than the genome-wide average (*P* < 10^−199^ and *P* = 0 for *A. oxysepala* var. *kansuensis* and *A. coerulea*, respectively) provides further support for this possibility. Nevertheless, 36.2% (815/2250) of the species-specific genes in *A. oxysepala* var. *kansuensis* have Gene Ontology (GO) assignments, compared with only 18.6% (951/5119) in *A. coerulea*, suggesting that our gene set is of higher confidence. In fact, our annotation is supported by much stronger evidence since we applied both Iso-Seq and RNA-Seq (PE150) data from different tissue samples (Table S8) to annotate the *A. oxysepala* var. *kansuensis* genome, in contrast to the annotation of *A. coerulea*, for which only RNA-Seq (SE35 and SE40) from sepals of this species but numerous sequencing data (mostly SE51) from other species were used. The fact that 97.2% of the genes in *A. oxysepala* var. *kansuensis* show homology with functionally annotated genes reaffirms the high confidence of our gene set. All these results show that we obtained a high-quality genome that will pave the way for spur reduction, adaptation, and evolutionary studies of *Aquilegia*.

### Genome divergence between *A. oxysepala* var. *kansuensis* and *A. coerulea*

It is interesting that the two *Aquilegia* species show considerable differences in their genomes. First, there has been a translocation between chromosomes 1 (A.ox_chr1) and 4 (A.ox_chr4) in *A. oxysepala* var. *kansuensis* relative to *A. coerulea*. Previously, it was observed that Chr4 in two other *Aquilegia* and one *Semiaquilegia* species have similar organizational patterns^10^, suggesting that this chromosome might have a conserved structure. One important piece of evidence is that 5S rDNA loci are uniquely localized to Chr4 in all three species^10^. We found that in *A. oxysepala* var. *kansuensis*, 5S rDNA loci are located on both A.ox_chr1 and A.ox_chr4, the majority of which are on the segments that are homologous to Chr4 of *A. coerulea* (Aco_chr4) (Table S14). Thus, it is likely that the translocation event was specific to *A. oxysepala* var. *kansuensis*. More interestingly, we also found 5S rDNA loci on other segments, including the 2,542,608–2,542,670 and 15,215,141–15,215,260 regions of A.ox_chr1 and the 33,260,556–33,260,618 region of A.ox_chr4 (Table S14). This indicates the possibility that the 5S rDNA was not unique to Aox_chr4 even before the chromosome translocation. Second, the two genomes are different in the number and content of annotated protein-coding genes, transposable elements, and noncoding RNAs. Particularly, among protein-coding genes, only 68.0% (17,391/25,571) of the genes of *A. oxysepala* var. *kansuensis* are one-to-one orthologous to genes of *A. coerulea*, which means that numerous orthologs are different in copy number. Third, it has been reported that the sequence divergence between species of *Aquilegia* from different geographic regions (such as Asia and North America) is at least 0.81%^10^. Since a divergence level as low as 0.15% would lead to marked biases in some genetic/genomic analyses^32^, using either one of the genomes as the reference for the other would be unsatisfactory. These results demonstrate the evolution of chromosomes in the plant genome and further highlight the importance of sequencing the genome of *A. oxysepala* var. *kansuensis*.

### Special evolutionary pattern of Chr4 of *A. oxysepala* var. *kansuensis*

It has been shown that the entire Chr4 of *A. coerulea* (A.co_chr4) has evolved uniquely under reduced purifying selection, demonstrating a “decaying” nature^31^ with a much higher level of polymorphism and lower gene density than other chromosomes^10^. Moreover, it is suggested that this evolutionary pattern of Chr4 began before the split of *Aquilegia* and *Semiaquilegia*^10^. In concordance with this, we found that the chromosome segments in *A. oxysepala* var. *kansuensis* (A.ox_chr4_1 and A.ox_chr1_2) that are homologous to A.co_chr4 have a significantly lower gene density. What is surprising is that A.ox_chr4_1 and A.ox_chr1_2 do not show the same pattern in their *d*_N_/*d*_S_ ratios. A.ox_chr4_1 has a significantly higher *d*_N_/*d*_S_ ratio than other chromosomes or chromosome segments, while the difference for A.ox_chr1_2 is weaker. This indicates that the translocation of this segment to Chr1 might have influenced the evolution of genes therein so that they are under more functional constraints than before. In this case, it is tempting to speculate that the translocation of a segment (A.ox_chr4_2) from Chr1, which is likely evolving under high functional constraint (high gene density and low *d*_N_/*d*_S_ ratio), might also have an impact on the “decay” of Chr4. More genomic data from other *Aquilegia* taxa would be helpful to further clarify this influence.

## Materials and methods

### Plant material collection, DNA extraction, and sequencing

We excavated whole plants of *A. oxysepala* var*. kansuensis* from a population in Yuzhong County, Gansu Province, China (N35°47′33″, E104°3′12″), and cultivated them in a growth chamber. Fresh young leaves of an individual were collected, and genomic DNA was extracted using the TIANGEN DNAsecure Plant Kit (GP1). For short-read sequencing, an ~350 bp insert size pair-end library was constructed and sequenced using the Illumina HiSeq 4000 platform. A total of 18.3 Gb raw data were generated (Table S1). For long-read sequencing, ~20 kb SMRTbell libraries were prepared and sequenced using PacBio Sequel Sequencer, which produced 37.6 Gb raw data (Table S3).

To extract enough DNA for the construction of optical mapping libraries, we collected young, fresh leaves from dark-treated seedlings of *A. oxysepala* var. *kansuensis*. High-molecular-weight DNAs were then isolated using the BioNano Prep Plant Tissue DNA Isolation Kit Contents (Part # 80003). Direct Labeling Enzyme 1 (DLE-1) was used to digest the DNAs, which were then fluorescently labeled, stained, and loaded onto a Saphyr Chip for sequencing. Nearly 301 Gb optical mapping data were generated. For the Hi–C data, we constructed two libraries, which were subjected to sequencing on the Illumina HiSeq 4000 platform, yielding a total of 36.3 Gb data (Table S5).

### Genome assembly

PacBio SMRT long reads were assembled using Falcon^33^ (Branch 3.1) (--max_diff 100 --max_cov 100 --min_cov 2 --min_len 5000) after self-correction. The resulting contigs were then polished by Quiver^34^ using the long reads. SSPACE-LongRead^35^ was applied to merge the contigs into scaffolds with default parameters. Finally, Illumina reads were mapped back to polish the scaffolds using Pilon (—threads 20 —frags)^36^.

Genome map assemblies for *A. oxysepala* var. *kansuensis* were generated using Bionano Solve Pipeline version 3.3 and Bionano Access version 1.3.0 (https://bionanogenomics.com/support/software-downloads/). Low-quality optical molecules with length ≤ 180 kb or label number ≤9 were removed. A rough assembly was first performed with the following parameters: -i 0 -V 0 -A -z -u –m. A second assembly, using the first round result as reference, was launched with the following parameters: -y -r (rough assembly cmap) -V 0 -m. To create hybrid scaffolds, optical maps were aligned to PacBio-assembled contigs and scaffolded with BioNano’s hybrid-scaffold tool. The process included comparing the BioNano genome nick-based maps to the in silico nick maps of the genome sequence to find their best matches and potential reciprocal scaffolding of each dataset. If there were conflicts between the sequence and optical maps, both of them were cut at the conflict sites and assembled again with the hybrid-scaffold parameter “-B 2 -N 2”.

To construct chromosome-level assemblies, we further applied the 36.3 Gb Hi–C data. Almost 31.9 Gb clean data were retained after removing adapter sequences and low-quality reads, i.e., those with a ratio of N higher than 0.1 and/or quality value less than 5 (*Q* < 5). Unmapped reads, self-ligated reads, dangling-end reads, internal fragment reads and reads with incorrect sizes were further removed using HiCUP software^37^. Hi–C reads were mapped to the hybrid scaffolds, and Hi–C contact frequency between genomic loci was computed using Juicer (version 1.7.6)^26^. 3D-DNA (version 180114)^21^ was used to anchor and orient scaffolds based on the contact frequency calculated from mapped Hi–C read pairs to obtain the pseudomolecules for two rounds with default parameters. During this process, we manually corrected the misassembled order, oriented scaffolds of DNA based on Hi–C data, and took advantage of the telomere-to-telomere contact enrichment associated with genomes in the Rabl configuration to obtain seven pseudochromosomes using Juicebox Assembly Tools (JBAT version 1.8.8)^38^. The Hi–C read contact frequency matrix was visualized using Juicebox (version 1.8.8)^38^.

### Genome annotation

Two methods (i.e., homology alignment and de novo annotation) were used to extract the repeats in the genome of *A. oxysepala* var. *kansuensis*. The repeats in a plant genome can be divided into tandem repeats and interspersed repeats. For tandem repeats, the software TRF version 4.09 (http://tandem.bu.edu/trf/trf.html) was used to make de novo predictions. For the prediction of transposable elements, two approaches were used: the first was to search against the Repbase database (http://www.girinst.org/repbase) using RepeatMasker (v4.0.5)^39^ with default parameters, and the second was de novo prediction through LTR_FINDER (v1.0.7) (http://tlife.fudan.edu.cn/ltr_finder/), RepeatScout (v1.0.5) (http://www.repeatmasker.org/), and RepeatModeler (v1.0.3) (http://www.repeatmasker.org/) with default parameters.

Noncoding RNAs (ncRNAs) include tRNA, rRNA, miRNA, and snRNA. tRNA was predicted by tRNAscan-SE^40^. rRNA was annotated by BLASTN searches against other species. miRNAs and snRNAs were identified by searching against the Rfam database (13.0)^41^ with default parameters using INFERNAL software (v1.1.2)^42^.

A combination of three methods, including ab initio prediction, homology-based prediction and transcriptome-assisted prediction, was used to identify protein-coding genes. For homology-based prediction, we used sequences of six species, including *A. coerulea*, *O. sativa*, *A. trichopoda*, and *Populus trichocarpa* from Phytozome (https://phytozome.jgi.doe.gov/); *A. thaliana* from TAIR (https://www.arabidopsis.org); and *P. somniferum* from communications with the relevant authors^30^. TBLASTN searches (*e*-value ≤ 1e−5) were then conducted against the *A. oxysepala* var. *kansuensis* assembly to identify homologous proteins, which were then aligned to the assembly by GeneWise (v2.4.1)^43^ to annotate gene structures. For ab initio prediction, we employed Augustus (v3.2.3)^44^, Geneid (v1.4)^45^, Genescan (v1.0)^46^, GlimmerHMM (v3.04)^47^, and SNAP (v2013.11.29)^48^ software with default parameters. We used both Iso-Seq and RNA-Seq datasets from different tissue samples (i.e., roots, stems, leaves, flowers, fruits, and seeds) at different stages of development as evidence for gene annotation. We constructed five Iso-Seq libraries, which contained RNAs isolated from five samples, respectively, i.e., leaves of different stages, stems+roots, seedlings, small inflorescences (with flowers of early stages), and large inflorescences (with flowers of medium-late stages and seeds). These libraries were then sequenced on the PacBio Sequel platform, yielding a total of 47.6 Gb raw data. RNA-Seq data were generated by pooling equal amounts of RNA obtained from the above five samples and sequencing on the Illumina HiSeq 4000 platform. RNA-Seq raw reads were filtered and mapped to our genome assembly using TopHat (v2.0.11)^49^ to identify exon regions and splice positions. The alignment results were then input into Cufflinks (v2.2.1)^50^ with default parameters for genome-based transcript assembly. Iso-Seq data were processed through the standard Iso-Seq pipeline. The generated transcripts, together with the genome-guided assembly of RNA-Seq data, were integrated with the Program to Assemble Spliced Alignments (PASA)^51^. A nonredundant reference gene set was generated by merging genes predicted by the three aforementioned methods with EVidenceModeler (EVM, v1.1.1)^51^ and then updated using PASA^51^. Functions were assigned to each gene according to its best matches by aligning its protein sequence to the Swiss-Prot and NR databases using BLASTP (*e*-value ≤ 1e−5). Motifs and domains were annotated using InterProScan70 (v5.31)^52^ by searching against publicly available databases, including ProDom, PRINTS, Pfam, SMRT, PANTHER, and PROSITE. The GO terms for each gene were assigned according to the corresponding InterPro entry.

### Genome alignment and microsynteny detection

We used Nucmer, contained in the MUMmer package version 3.23^53^, to align the genomes of the two species, with default parameters. The results were then filtered by delta-filter program with “-i 90 -g –q”. To infer gene-level synteny, we used BLASTP (-evalue 1e−20 -num_threads 16 -outfmt 6) to generate protein alignment between the two species. The outputs were then imported into MCScanX^54^ to identify syntenic blocks.

### Orthogroup and gene family classification

OrthoFinder^27^ (orthofinder -f fastadata -S blast -M msa -T raxml) software was used to group genes of different species, and in-house R scripts were applied to count the number of ortholog clusters obtained. Based on gene family information of *A*. *coerulea* (http://www.supfam.org/genome/Ac), we performed BLASTP (-gapopen 11 -gapextend 1 -max_target_seqs 5) searches of each *A. oxysepala* var. *kansuensis* sequence against the proteome of *A. coerulea* and assigned it to a certain gene family based on the best hit.

### Phylogenetic analysis of the MADS-box gene family

We first retrieved all MADS-box gene sequences classified by using the gene family information of *A*. *coerulea*. When aligning the protein sequences of these genes, we found five in *A. coerulea* and seven in *A. oxysepala* var. *kansuensis* that had incomplete MADS-box domains. We mapped these sequences to their corresponding genomic regions using TBLASTN and manually curated the annotations. All MADS-box protein sequences were aligned using the hmmalign program in the HMMER package version 3.0^55^ and the SRF-domain (MADS-box domain) model downloaded from Pfam (http://pfam.sanger.ac.uk/). The corresponding CDS sequence alignment was generated using PAL2NAL^56^. Nucleotide sites in the MADS-box domain were used for phylogenetic analysis, which was performed in MEGA7^57^ (pairwise deletion and *p*-distance model) with 1000 bootstrap replications.

### Gene density and *d*_N_/*d*_S_ analyses

Gene density was calculated for nonoverlapping 300 kb windows along the whole chromosome using in-house R scripts. Then the ggplot2 package^58^ in R version 3.6.2 was used for plotting, and the Wilcox test was employed for statistical analysis. One-to-one ortholog clusters of the two species identified using OrthoFinder were used for *d*_N_/*d*_S_ analysis. Protein sequences of each pair of orthologs were aligned using MUSCLE3.8.31^59^ software with default parameters. PAL2NAL^56^ was then applied to create the corresponding CDS sequence alignment, and trimAL software^60^ was used to remove alignment-ambiguous codons. Finally, the Perl script (dS_dN_MNG.PL) downloaded from the website (https://sites.google.com/view/masafumi-nozawa/scripts) was applied to calculate the *d*_N_ and *d*_S_ values using a modified Nei–Gojobori model, and in-house R scripts were used to calculate the *d*_N_/*d*_S_ ratios.

### Identification of collinear paralogs and calculation of *d*_S_ values

We used MCScanX^54^ software to identify synteny blocks within each of the *A*. *oxysepala* var. *kansuensis*, *A. coerulea*, and grape genomes. One-to-one orthologs between *A*. *oxysepala* var. *kansuensis* and grape were obtained by using OrthoFinder^27^. The *d*_S_ value for each pair of genes was then calculated as described above using the modified Nei–Gojobori model. The geom_density^57^ function in R was used to compute and draw kernel density estimates of each *d*_S_ list.

## Supplementary information

Supplementary information
