# Supplementary material for: A chromosome-scale reference genome of Aquilegia oxysepala var. kansuensis
Source: Hortic Res. 2020 Jul 1;7:113. doi: 10.1038/s41438-020-0328-y (PMC7326910; doi:10.1038/s41438-020-0328-y)
Supplement: Supplementary file 1 — Supplementary information [file 41438_2020_328_MOESM1_ESM.docx]

**Supplementary information**

**Figures:**

**Figure S1. Genome size estimation of *A. oxysepala* var. *kansuensis* (A.ox) by using flow cytometry with *Arabidopsis* *thaliana* as reference.** The main peak of *Arabidopsis* and *A. oxysepala* var. *kansuensis* is 18.9 and 47.3, respectively. According to the formula “peak (ref) / genome size (ref) = peak (A.ox) / genome size (A.ox)”, we estimated that the genome size of A.ox is 312 Mb.


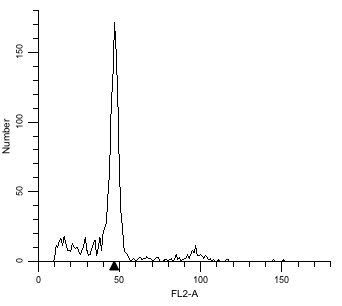


FL2-A

0

50

100

150

Number

0

50

100

150

**Figure S2. Distribution of 17-mer frequency.** A total of 18.2 Gb high-quality short reads were used to generate the 17-mer depth distribution curve frequency information.

Depth

Frequency

**Figure S3. Coverage distribution of PacBio long reads to the *A. oxysepala* var. *kansuensis* genome.** Each blue point in the panel designates the coverage in a 100 kb window. Red arrows indicate breakpoints.


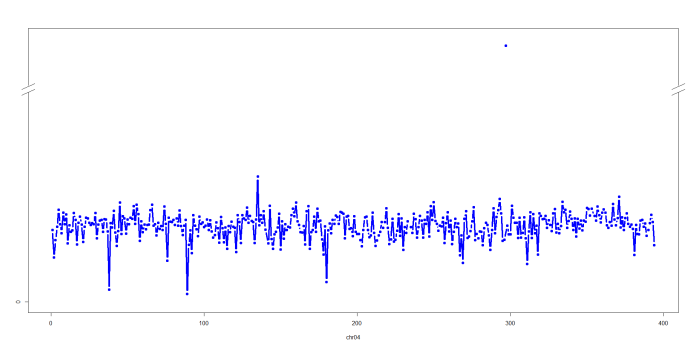


A.ox_chr4


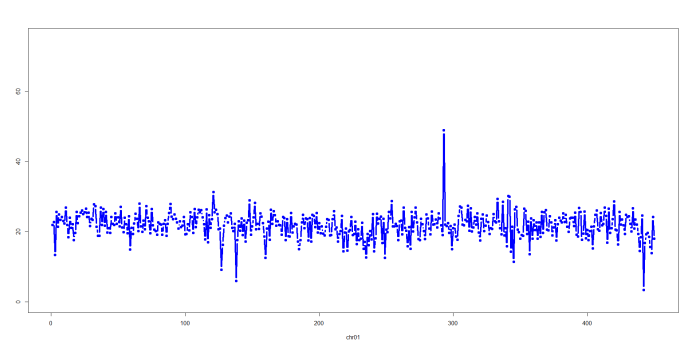


A.ox_chr1

0

100

200

300

400

0

20

40

60


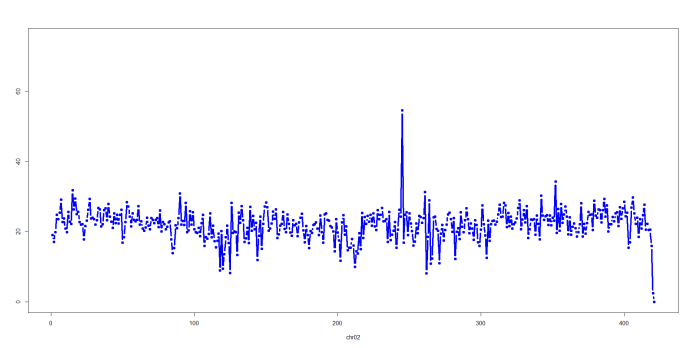


A.ox_chr2

0

100

200

300

400

0

20

40

60


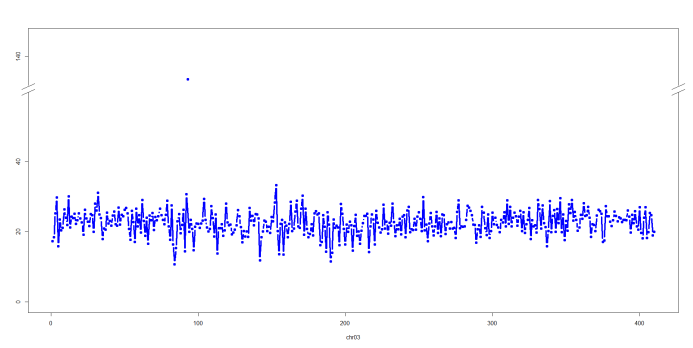


A.ox_chr3

0

100

200

300

400

0

20

40

140

0

100

200

300

400

0

20


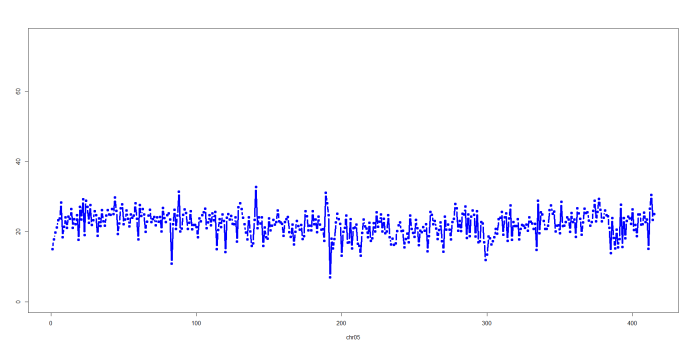


A.ox_chr5

0

100

200

300

400

0

20

40

60


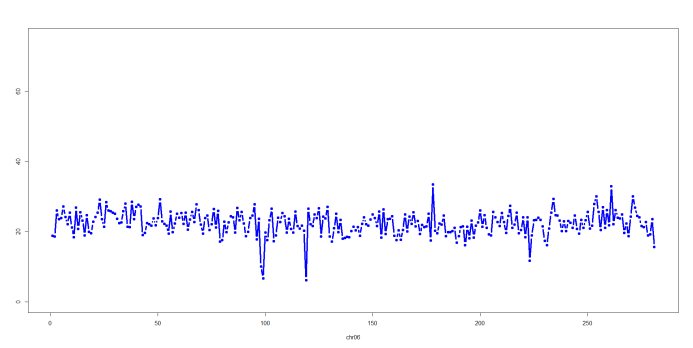


A.ox_chr6

0

50

100

150

200

0

20

40

60

250


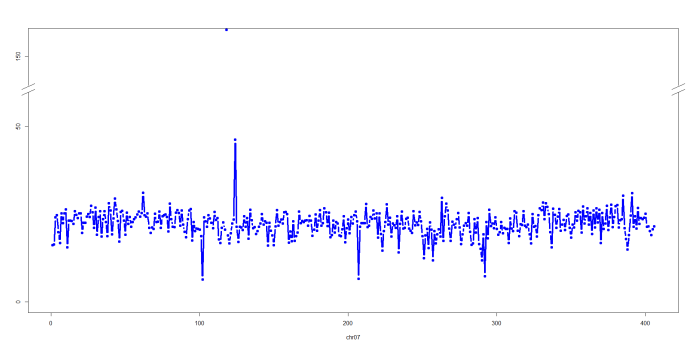


A.ox_chr7

0

100

200

300

400

0

50

150

**Figure S4. Confirmation of the assembly using Hi-C data.** **a.** Hi-C heatmap of the *A. oxysepala* var. *kansuensis* genome assembly. **b.** A heatmap generated by mapping the Hi-C data to the *A. oxysepala* var. *kansuensis* genome with two hypothesized chromosome 1 and 4 (h_A.ox_chr1 and h_A.ox_chr4), which are created by artificially moving back the translocated segments. **c.** A heatmap generated by mapping the Hi-C data to the *A. coerulea* genome*.* Apparent conflicts between h_A.ox_chr1 and h_A.ox_chr4, as well as between A.co_chr1 and A.co_chr4 could be observed (shown by blue arrow), indicating the structural variation between these two genomes.


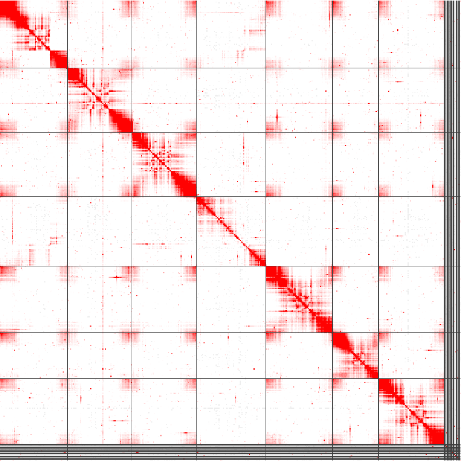


A.co_chr1

A.co_chr2

A.co_chr3

A.co_chr4

A.co_chr5

A.co_chr6

A.co_chr7

A.co_chr1

A.co_chr2

A.co_chr3

A.co_chr4

A.co_chr5

A.co_chr6

A.co_chr7

A.ox_chr1

A.ox_chr2

A.ox_chr3

A.ox_chr4

A.ox_chr5

A.ox_chr6

A.ox_chr7

A.ox_chr1

A.ox_chr2

A.ox_chr3

A.ox_chr4

A.ox_chr5

A.ox_chr6

A.ox_chr7


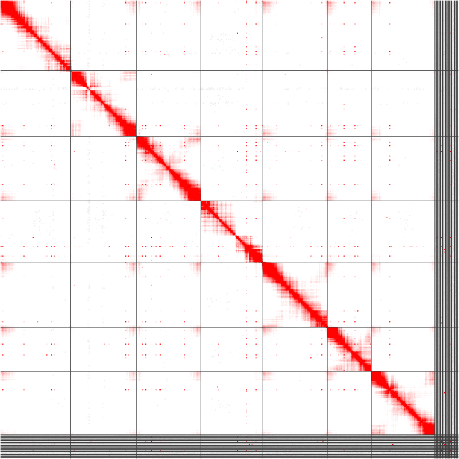

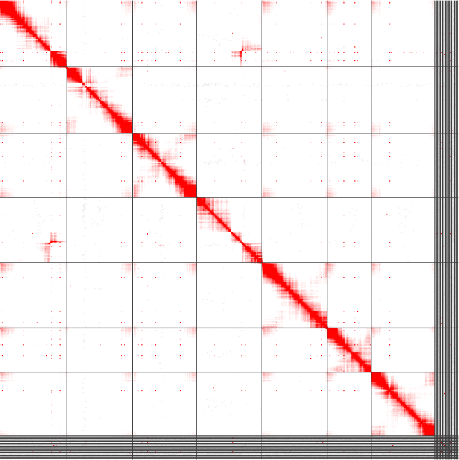


h_A.ox_chr1

A.ox_chr2

A.ox_chr3

h_A.ox_chr4

A.ox_chr5

A.ox_chr6

A.ox_chr7

h_A.ox_chr1

A.ox_chr2

A.ox_chr3

h_A.ox_chr4

A.ox_chr5

A.ox_chr6

A.ox_chr7

**a**

**b**

**c**

**Figure S5. Comparison of LAI scores between *A. oxysepala* var*. kansuensis* and *A. coerulea* along the seven chromosomes.** LAI scores in genomic regions of *A. oxysepala* var. *kansuensis* and *A. coerulea* are compared within a 3 Mb block per 300 kb step.


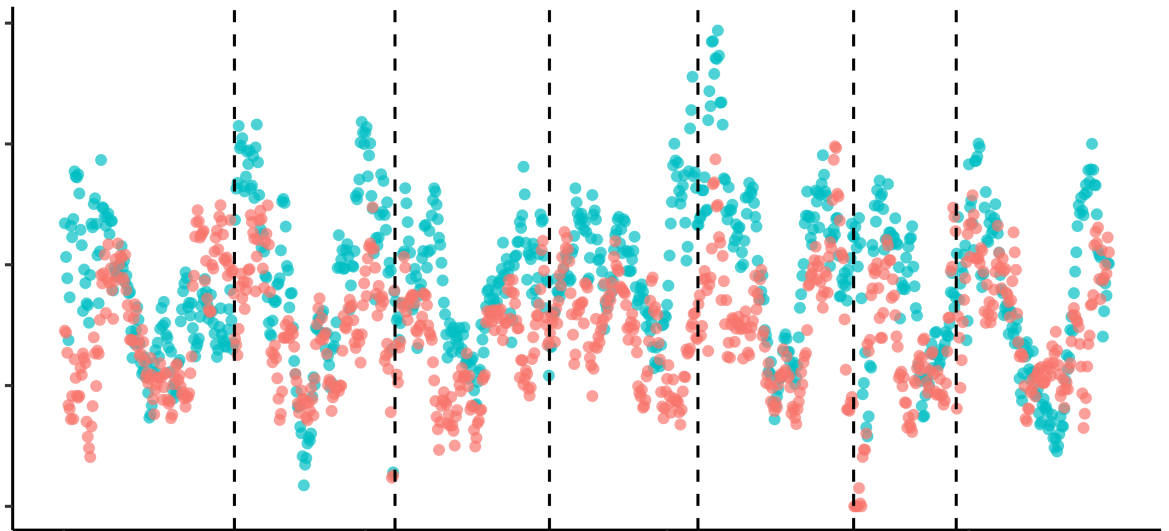


0

10

20

30

40

chr1

chr2

chr3

chr4

chr5

chr6

chr7


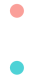


A.co

A.ox

**Figure S6. Comparison of exon number, gene length, CDS length and exon length between *A. oxysepala* var*. kansuensis* and other species.**


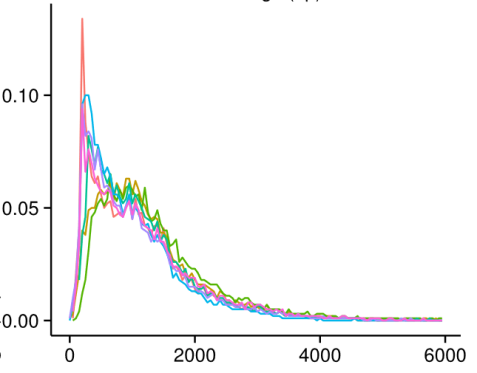

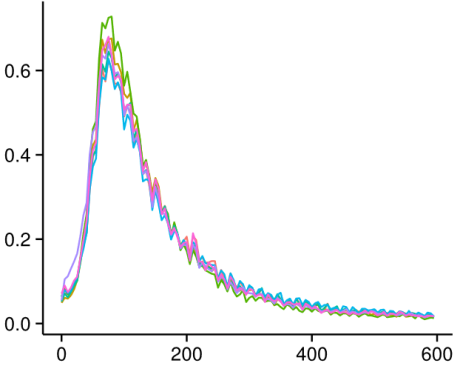

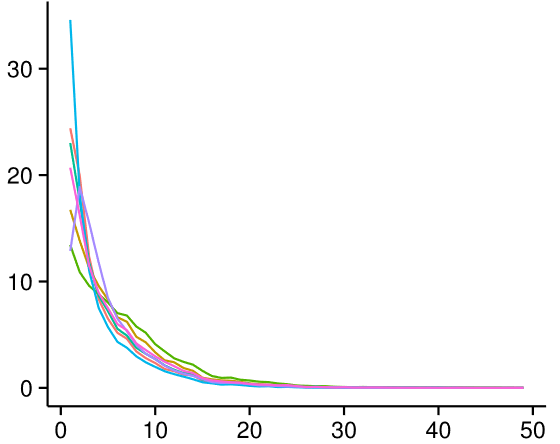

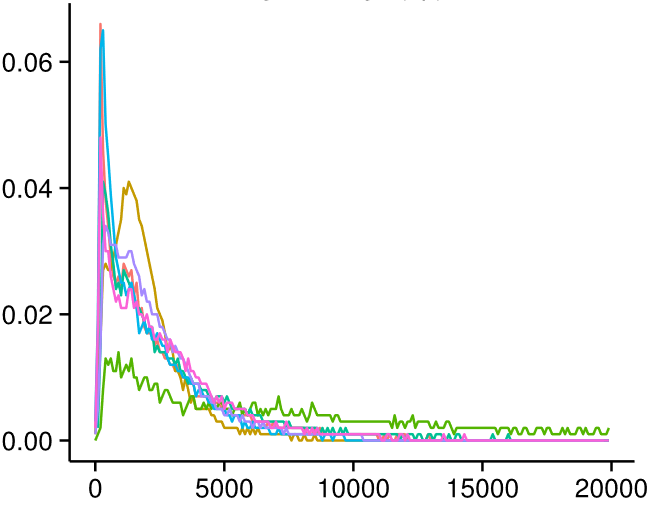


Exon number

Gene length (bp)

CDS length (bp)

Exon length (bp)

Percentage in genome (%)

Percentage in genome (%)

Percentage in genome (%)

Percentage in genome (%)


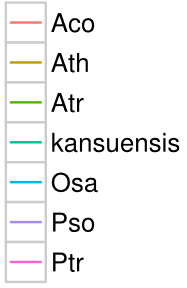


*A. coerulea*

*Arabidosis thaliana*

*Amborella trichopoda*

*A. oxysepala var. kansuensis*

*Oryza sativa*

*Papaver somniferum*

*Populus trichocarpa*

**
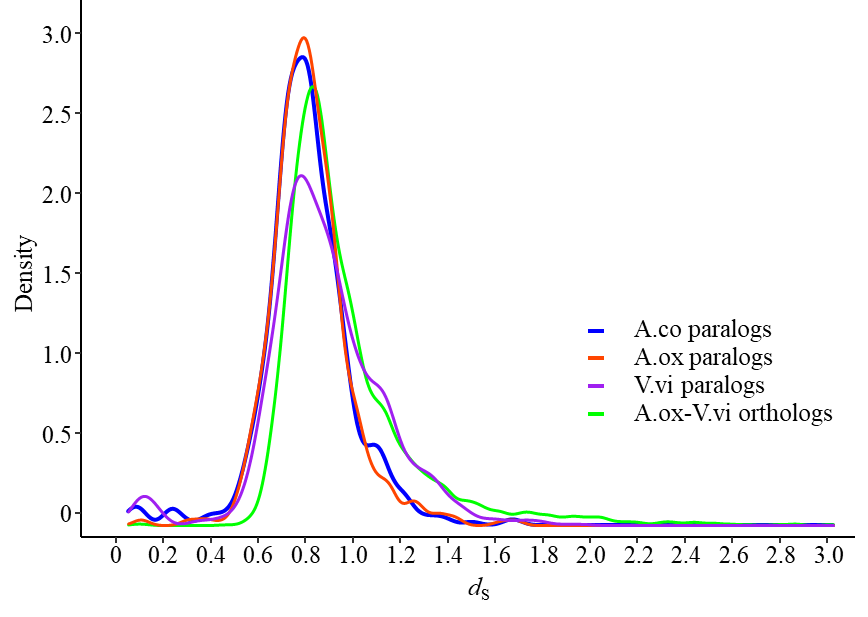
**

**Figure S7. Synonymous substitution rates (*d*_S_) between collinear paralogs in each of *A. oxysepala* var*. kansuensis*, *A*. *coerulea* and grape (*Vitis vinifera*), and between the one-to-one orthologs of *A. oxysepala* var*. kansuensis* and grape (*d*_S_ higher than 3.0 were not shown).**

**Tables:**

**Table S1.** Summary of library construction and sequencing of Illumina data.

| **Library** | **Insert size (bp)** | **Average read length (bp)** | **Raw data size (Gb)** | **Sequence coverage**^1^ **(×)** |
| --- | --- | --- | --- | --- |
| Paired-end reads | 350 | 150 | 18.3 | ~52 |

^1^Depth is calculated under the estimate of a genome size of 349 Mb.

**Table S2.** Estimation of the *A. oxysepala* var. *kansuensis* genome size using 17 K-mer analysis.

| **Clean base**  **(Gp)** | **K-mer** | **K-mer**  **number** | **K-mer depth** | **Estimated genome**  **size (Mb)** | **Heterozygous**  **rate (%)** | **Repeat rate (%)** |
| --- | --- | --- | --- | --- | --- | --- |
| 18.2 | 17 | 15,224,138,248 | 43 | 349.72 | 0.15 | 52.15 |

**Table S3.** Summary of PacBio long-read sequencing data.

| **Insert size (Kb)** | **Average read length (Kb)** | **Raw data size (Gb)** | **Sequence coverage**^1^ **(×)** |
| --- | --- | --- | --- |
| ~20 | 17 | 37.6 | ~105 |

^1^Depth is calculated under the estimate of a genome size of 349 Mb.

**Table S4.** Statistics of BioNano optical DLE-1 molecules.

| **BioNano optical DLE-1 molecules statistics** | **Statistics** |
| --- | --- |
| Total number of molecules（filter to >180Kb) | 802,265 |
| Total length (Mb) | 244,498.8 |
| Average length (Kb) | 304.8 |
| Molecule N50 (Kb) | 310.2 |
| Label density (/100Kb) | 10.9 |
| Raw coverage of reference (×) | 724.2 |
| **Molecules aligned to reference:** |  |
| Total number of aligned molecules | 374,793 |
| Fraction of aligned molecules | 0.47 |
| Effective coverage of reference (×) | 292.6 |
| Average confidence | 28.6 |

**Table S5.** Summary of Hi-C data.

| **Sample NO.** | **Insert size (bp)** | **Raw data (Gb)** | **Clean data (Gb)** | **% effect rate** |
| --- | --- | --- | --- | --- |
| Library 1 | 350 | 15.2 | 15.1 | 46.18 |
| Library 2 | 350 | 21.1 | 16.8 | 46.87 |

**Table S6.** Statistics of contigs and scaffolds of the primary genome assembly based on PacBio data.

|  | **Length** | | **Number** | |
| --- | --- | --- | --- | --- |
|  | **Contig (bp)** | **Scaffold (bp)** | **Contig** | **Scaffold** |
| Total | 292,235,404 | 292,426,027 | 852 | 738 |
| Max | 7,819,963 | 7,827,536 | - | - |
| Number≥2000 | - | - | 782 | 674 |
| N50 | 2,220,702 | 2,606,950 | 39 | 34 |
| N60 | 1,710,843 | 1,970,130 | 54 | 48 |
| N70 | 1,339,781 | 1,430,819 | 74 | 65 |
| N80 | 911,308 | 1,100,109 | 100 | 88 |
| N90 | 454,562 | 524,975 | 144 | 125 |

**Table S7.** Alignment statistics of the BioNano genome maps to the *A. oxysepala* var. *kansuensis* genome assembly.

| **BioNano genome maps** | **Statistics** |
| --- | --- |
| Number genome maps | 34 |
| Total genome map length (Mb) | 308.97 |
| Mean genome map length (Mb) | 9.09 |
| Genome map N50 (Mb) | 20.42 |
| Total reference length (Mb) | 293.21 |
| Total genome map length/reference length | 1.05 |
| Total number of aligned genome maps | 25 (0.74) |
| Total aligned length (Mb) | 283.94 |
| Total aligned length / reference length | 0.97 |
| Total unique aligned length (Mb) | 277.34 |
| Total unique aligned length / reference length | 94.6% |

**Table S8.** Statistics of RNA-Seq and Iso-Seq data.

|  | **Tissues** | **Read length (mean)** | **Raw data (Gb)** | **Clean data (Gb)** |
| --- | --- | --- | --- | --- |
| PacBio | Small inflorescence | 2.91Kb | 4.37 | 4.29 |
|  | Large inflorescence | 3.01Kb | 9.59 | 9.40 |
|  | Leaves | 2.92Kb | 6.55 | 6.44 |
|  | Stems+Roots | 2.46Kb | 10.20 | 9.98 |
|  | Seedlings | 3.83Kb | 16.90 | 16.63 |
| Illumina | Pooling | 150bp | 12.97 | 12.77 |

**Table S9.** Copy number comparison in each gene family.

| **Family Name** | **Number of genes in A.ox** | **Number of genes in A.co** |
| --- | --- | --- |
| P-loop containing nucleoside triphosphate hydrolases | 1033 | 1022 |
| Protein kinase-like (PK-like) | 824 | 857 |
| F-box domain | 422 | 444 |
| RING or U-box | 404 | 398 |
| L domain-like | 352 | 439 |
| ARM repeat | 337 | 299 |
| TPR-like | 336 | 348 |
| Cytochrome P450 | 333 | 419 |
| NAD(P)-binding Rossmann-fold domains | 324 | 353 |
| alpha or beta-Hydrolases | 284 | 285 |
| RNI-like | 267 | 255 |
| Homeodomain-like | 266 | 260 |
| WD40 repeat-like | 250 | 227 |
| S-adenosyl-L-methionine-dependent methyltransferases | 247 | 247 |
| RNA-binding domain, RBD | 242 | 225 |
| Thioredoxin-like | 215 | 223 |
| MFS general substrate transporter | 200 | 191 |
| (Trans)glycosidases | 188 | 202 |
| UDP-Glycosyltransferase or glycogen phosphorylase | 172 | 206 |
| HAD-like | 160 | 128 |
| Ribonuclease H-like | 147 | 106 |
| Clavaminate synthase-like | 144 | 143 |
| EF-hand | 144 | 133 |
| Nucleotide-diphospho-sugar transferases | 135 | 114 |
| Winged helix DNA-binding domain | 132 | 125 |
| Nucleic acid-binding proteins | 128 | 109 |
| FAD or NAD(P)-binding domain | 121 | 113 |
| Pectin lyase-like | 116 | 111 |
| Ankyrin repeat | 115 | 103 |
| FYVE or PHD zinc finger | 110 | 91 |
| beta-beta-alpha zinc fingers | 107 | 94 |
| HCP-like | 105 | 120 |
| Chaperone J-domain | 103 | 104 |
| HLH, helix-loop-helix DNA-binding domain | 103 | 102 |
| DNA-binding domain | 101 | 96 |
| Cysteine proteinases | 98 | 88 |
| Multidrug resistance efflux transporter EmrE | 92 | 82 |
| Actin-like ATPase domain | 91 | 63 |
| Cupredoxins | 85 | 87 |
| Ubiquitin-like | 85 | 103 |
| C2 domain (Calcium or lipid-binding domain, CaLB) | 84 | 75 |
| Bet v1-like | 81 | 94 |
| SGNH hydrolase | 80 | 88 |
| Concanavalin A-like lectins or glucanases | 79 | 75 |
| POZ domain | 78 | 93 |
| DNA-binding pseudobarrel domain | 75 | 74 |
| PLP-dependent transferases | 75 | 71 |
| ABC transporter transmembrane region | 69 | 63 |
| GST C-terminal domain-like | 67 | 74 |
| Nucleotidylyl transferase | 67 | 40 |
| Bifunctional inhibitor or lipid-transfer protein or seed storage 2S albumin | 66 | 92 |
| Acyl-CoA N-acyltransferases (Nat) | 65 | 51 |
| Acylphosphatase or BLUF domain-like | 65 | 51 |
| Acid proteases | 64 | 67 |
| Mitochondrial carrier | 64 | 56 |
| UBC-like | 63 | 53 |
| Ribosomal protein S5 domain 2-like | 62 | 59 |
| PP2C-like | 61 | 55 |
| Metallo-dependent phosphatases | 60 | 56 |
| Plant invertase or pectin methylesterase inhibitor | 60 | 49 |
| Subtilisin-like | 58 | 65 |
| NAC domain | 57 | 62 |
| Retrovirus zinc finger-like domains | 57 | 41 |
| Terpenoid cyclases or Protein prenyltransferases | 57 | 57 |
| alpha-D-mannose-specific plant lectins | 56 | 74 |
| Glucocorticoid receptor-like (DNA-binding domain) | 55 | 48 |
| Terpenoid synthases | 55 | 64 |
| Heme-dependent peroxidases | 54 | 81 |
| HSP20-like chaperones | 54 | 59 |
| ATPase domain of HSP90 chaperone or DNA topoisomerase II or histidine kinase | 52 | 41 |
| HMA, heavy metal-associated domain | 52 | 45 |
| Kelch motif | 52 | 55 |
| CAD & PB1 domains | 51 | 48 |
| E set domains | 50 | 47 |
| Histone-fold | 50 | 74 |
| SET domain | 50 | 43 |
| SRF-like | 50 | 59 |
| Cyclin-like | 49 | 44 |
| Translation proteins | 49 | 43 |
| Adenine nucleotide alpha hydrolases-like | 48 | 48 |
| PUA domain-like | 48 | 36 |
| Galactose oxidase, central domain | 47 | 57 |
| N-terminal nucleophile aminohydrolases (Ntn hydrolases) | 47 | 43 |
| PH domain-like | 47 | 44 |
| Calcium ATPase, transduction domain A | 46 | 38 |
| Calcium ATPase, transmembrane domain M | 46 | 38 |
| UBA-like | 45 | 37 |
| CCCH zinc finger | 44 | 40 |
| vWA-like | 44 | 36 |
| Leucine zipper domain | 42 | 45 |
| Acetyl-CoA synthetase-like | 41 | 38 |
| Class II aaRS and biotin synthetases | 41 | 34 |
| GroES-like | 41 | 48 |
| Periplasmic binding protein-like II | 40 | 40 |
| RmlC-like cupins | 40 | 61 |
| ACT-like | 37 | 36 |
| DNase I-like | 37 | 31 |
| ENTH or VHS domain | 37 | 32 |
| Galactose-binding domain-like | 37 | 38 |
| Voltage-gated potassium channels | 37 | 27 |
| Eukaryotic type KH-domain (KH-domain type I) | 36 | 35 |
| Heat shock protein 70kD (HSP70), peptide-binding domain | 36 | 23 |
| Six-hairpin glycosidases | 36 | 37 |
| WRKY DNA-binding domain | 36 | 33 |
| FAD-linked reductases, C-terminal domain | 35 | 30 |
| Thiolase-like | 35 | 33 |
| Cullin repeat-like | 34 | 29 |
| ClpP or crotonase | 33 | 35 |
| SNARE-like | 33 | 34 |
| (Phosphotyrosine protein) phosphatases II | 32 | 33 |
| 6-phosphogluconate dehydrogenase C-terminal domain-like | 32 | 28 |
| Barwin-like endoglucanases | 32 | 33 |
| Anticodon-binding domain of a subclass of class I aminoacyl-tRNA synthetases | 31 | 17 |
| CBS-domain pair | 31 | 29 |
| Heat shock protein 70kD (HSP70), C-terminal subdomain | 31 | 20 |
| NTF2-like | 31 | 29 |
| Quinoprotein alcohol dehydrogenase-like | 31 | 32 |
| CRAL or TRIO domain | 30 | 29 |
| Sm-like ribonucleoproteins | 30 | 26 |
| SNARE fusion complex | 30 | 29 |
| cAMP-binding domain-like | 29 | 23 |
| Glyceraldehyde-3-phosphate dehydrogenase-like, C-terminal domain | 29 | 23 |
| GroEL apical domain-like | 29 | 24 |
| Phosphoenolpyruvate or pyruvate domain | 29 | 23 |
| Tudor or PWWP or MBT | 29 | 22 |
| Aldolase | 28 | 26 |
| Aquaporin-like | 28 | 26 |
| CheY-like | 28 | 26 |
| Cyclophilin-like | 28 | 28 |
| Nucleotidyltransferase | 28 | 25 |
| TRAF domain-like | 28 | 29 |
| Chlorophyll a-b binding protein | 27 | 33 |
| Galactose mutarotase-like | 27 | 31 |
| Periplasmic binding protein-like I | 27 | 28 |
| Bromodomain | 26 | 24 |
| CRAL or TRIO N-terminal domain | 26 | 25 |
| FtsH protease domain-like | 26 | 19 |
| GroEL equatorial domain-like | 26 | 20 |
| GroEL-intermediate domain like | 26 | 20 |
| Metal cation-transporting ATPase, ATP-binding domain N | 26 | 23 |
| Ribokinase-like | 26 | 25 |
| BRCT domain | 25 | 22 |
| Class I glutamine amidotransferase-like | 25 | 21 |
| FabD or lysophospholipase-like | 25 | 23 |
| Ferredoxin reductase-like, C-terminal NADP-linked domain | 25 | 23 |
| FKBP-like | 25 | 24 |
| Glycerol-3-phosphate (1)-acyltransferase | 25 | 22 |
| NAD(P)-linked oxidoreductase | 25 | 23 |
| Nudix | 25 | 23 |
| Osmotin, thaumatin-like protein | 25 | 24 |
| PHL pollen allergen | 25 | 23 |
| Translational machinery components | 25 | 22 |
| Tropomyosin | 25 | 19 |
| Zn-dependent exopeptidases | 25 | 23 |
| ALDH-like | 24 | 20 |
| dsRNA-binding domain-like | 24 | 25 |
| GAT-like domain | 24 | 19 |
| Glutathione synthetase ATP-binding domain-like | 24 | 22 |
| Metallo-hydrolase or oxidoreductase | 24 | 22 |
| PAZ domain | 24 | 26 |
| Ran binding protein zinc finger-like | 24 | 23 |
| Skp1 dimerisation domain-like | 24 | 42 |
| Glycosyl hydrolase domain | 23 | 23 |
| RCC1 or BLIP-II | 23 | 23 |
| Rhodanese or Cell cycle control phosphatase | 23 | 20 |
| Tubulin nucleotide-binding domain-like | 23 | 22 |
| DNA or RNA polymerases | 22 | 15 |
| DnaJ or Hsp40 cysteine-rich domain | 22 | 26 |
| Metalloproteases (zincins), catalytic domain | 22 | 27 |
| PRTase-like | 22 | 15 |
| Thiamin diphosphate-binding fold (THDP-binding) | 22 | 21 |
| Translation proteins SH3-like domain | 22 | 25 |
| Tubby C-terminal domain-like | 22 | 20 |
| Chromo domain-like | 21 | 18 |
| FAS1 domain | 21 | 22 |
| L30e-like | 21 | 16 |
| Ribulose-phoshate binding barrel | 21 | 16 |
| Tryptophan synthase beta subunit-like PLP-dependent enzymes | 21 | 16 |
| Tubulin C-terminal domain-like | 21 | 21 |
| ValRS or IleRS or LeuRS editing domain | 21 | 7 |
| Calcium-dependent phosphotriesterase | 20 | 16 |
| DPP6 N-terminal domain-like | 20 | 16 |
| FAD-binding or transporter-associated domain-like | 20 | 31 |
| HSP40 or DnaJ peptide-binding domain | 20 | 23 |
| PA domain | 20 | 19 |
| Single hybrid motif | 20 | 16 |
| YhbY-like | 20 | 15 |
| Aminoacid dehydrogenase-like, N-terminal domain | 19 | 17 |
| PDZ domain-like | 19 | 20 |
| Pseudouridine synthase | 19 | 17 |
| Riboflavin synthase domain-like | 19 | 18 |
| t-snare proteins | 19 | 18 |
| Double Clp-N motif | 18 | 11 |
| Lipase or lipooxygenase domain (PLAT or LH2 domain) | 18 | 16 |
| Phosphoglycerate mutase-like | 18 | 18 |
| Phosphohistidine domain | 18 | 18 |
| PLC-like phosphodiesterases | 18 | 18 |
| SMAD or FHA domain | 18 | 16 |
| ADP-ribosylation | 17 | 17 |
| AF0104 or ALDC or Ptd012-like | 17 | 18 |
| Class II aaRS ABD-related | 17 | 13 |
| Formin homology 2 domain (FH2 domain) | 17 | 17 |
| Rhomboid-like | 17 | 17 |
| Ypt or Rab-GAP domain of gyp1p | 17 | 14 |
| beta and beta-prime subunits of DNA dependent RNA-polymerase | 16 | 15 |
| FMN-linked oxidoreductases | 16 | 20 |
| HD-domain or PDEase-like | 16 | 14 |
| Metallo-dependent hydrolases | 16 | 15 |
| Thioesterase or thiol ester dehydrase-isomerase | 16 | 17 |
| Zinc beta-ribbon | 16 | 13 |
| At5g01610-like | 15 | 17 |
| CoA-dependent acyltransferases | 15 | 18 |
| EF-Tu or eEF-1alpha or eIF2-gamma C-terminal domain | 15 | 14 |
| Glyoxalase or Bleomycin resistance protein or Dihydroxybiphenyl dioxygenase | 15 | 13 |
| PAP or OAS1 substrate-binding domain | 15 | 13 |
| Ribonuclease Rh-like | 15 | 10 |
| SAICAR synthase-like | 15 | 15 |
| Smc hinge domain | 15 | 6 |
| Trypsin-like serine proteases | 15 | 16 |
| WW domain | 15 | 14 |
| Band 7 or SPFH domain | 14 | 14 |
| Beta-D-glucan exohydrolase, C-terminal domain | 14 | 19 |
| Cytochrome b5-like heme or steroid binding domain | 14 | 14 |
| Cytochrome b559 subunits | 14 | 14 |
| Formate or glycerate dehydrogenase catalytic domain-like | 14 | 15 |
| HMG-box | 14 | 14 |
| ISP domain | 14 | 11 |
| NagB or RpiA or CoA transferase-like | 14 | 13 |
| PreATP-grasp domain | 14 | 11 |
| SWIB or MDM2 domain | 14 | 14 |
| Trimeric LpxA-like enzymes | 14 | 15 |
| 2Fe-2S ferredoxin-like | 13 | 13 |
| alpha or beta knot | 13 | 12 |
| Calponin-homology domain, CH-domain | 13 | 12 |
| Carbamate kinase-like | 13 | 10 |
| Creatinase or aminopeptidase | 13 | 12 |
| EF-G C-terminal domain-like | 13 | 13 |
| HIT or MYND zinc finger-like | 13 | 13 |
| LysM domain | 13 | 15 |
| Phospholipase D or nuclease | 13 | 13 |
| PIN domain-like | 13 | 14 |
| PK beta-barrel domain-like | 13 | 12 |
| post-AAA+ oligomerization domain-like | 13 | 12 |
| Purple acid phosphatase, N-terminal domain | 13 | 14 |
| Acid phosphatase or Vanadium-dependent haloperoxidase | 12 | 11 |
| Activating enzymes of the ubiquitin-like proteins | 12 | 11 |
| Alkaline phosphatase-like | 12 | 11 |
| Arabinanase or levansucrase or invertase | 12 | 13 |
| Carbon-nitrogen hydrolase | 12 | 8 |
| DNA-glycosylase | 12 | 13 |
| GHMP Kinase, C-terminal domain | 12 | 12 |
| Hect, E3 ligase catalytic domain | 12 | 8 |
| HRDC-like | 12 | 10 |
| Lipoxigenase | 12 | 10 |
| PapD-like | 12 | 14 |
| PGBD-like | 12 | 13 |
| Prefoldin | 12 | 9 |
| Ribonuclease PH domain 2-like | 12 | 12 |
| Sec63 N-terminal domain-like | 12 | 8 |
| Starch-binding domain-like | 12 | 13 |
| Zn-binding ribosomal proteins | 12 | 18 |
| 14-3-3 protein | 11 | 14 |
| 4Fe-4S ferredoxins | 11 | 12 |
| Arginase or deacetylase | 11 | 10 |
| Bacterial exopeptidase dimerisation domain | 11 | 9 |
| Carbohydrate phosphatase | 11 | 11 |
| Cystatin or monellin | 11 | 10 |
| Dimeric alpha+beta barrel | 11 | 10 |
| DNA ligase or mRNA capping enzyme, catalytic domain | 11 | 8 |
| GYF domain | 11 | 10 |
| Homodimeric domain of signal transducing histidine kinase | 11 | 10 |
| LEA14-like | 11 | 11 |
| LuxS or MPP-like metallohydrolase | 11 | 11 |
| Mog1p or PsbP-like | 11 | 12 |
| NAD kinase or diacylglycerol kinase-like | 11 | 12 |
| Nop domain | 11 | 10 |
| Ribosomal protein L25-like | 11 | 7 |
| SBT domain | 11 | 10 |
| ACP-like | 10 | 11 |
| AN1-like Zinc finger | 10 | 9 |
| ArfGap or RecO-like zinc finger | 10 | 10 |
| Cation efflux protein transmembrane domain-like | 10 | 8 |
| Cryptochrome or photolyase, N-terminal domain | 10 | 9 |
| Cysteine-rich domain | 10 | 18 |
| Cytidine deaminase-like | 10 | 10 |
| GAF domain-like | 10 | 10 |
| Isocitrate or Isopropylmalate dehydrogenase-like | 10 | 10 |
| LexA or Signal peptidase | 10 | 9 |
| Lysozyme-like | 10 | 8 |
| PK C-terminal domain-like | 10 | 9 |
| Plus3-like | 10 | 8 |
| PR-1-like | 10 | 16 |
| Radical SAM enzymes | 10 | 11 |
| SAM or Pointed domain | 10 | 9 |
| 5' to 3' exonuclease, C-terminal subdomain | 9 | 10 |
| Actin depolymerizing proteins | 9 | 9 |
| Amidase signature (AS) enzymes | 9 | 8 |
| Ammonium transporter | 9 | 9 |
| BAR or IMD domain-like | 9 | 9 |
| beta-sandwich domain of Sec23 or 24 | 9 | 10 |
| BSD domain-like | 9 | 9 |
| Carbonic anhydrase | 9 | 11 |
| Cryptochrome or photolyase FAD-binding domain | 9 | 8 |
| D-aminoacid aminotransferase-like PLP-dependent enzymes | 9 | 9 |
| Di-copper centre-containing domain | 9 | 8 |
| DNA repair protein MutS, domain III | 9 | 7 |
| Ferritin-like | 9 | 8 |
| FMN-binding split barrel | 9 | 9 |
| Glutamine synthetase or guanido kinase | 9 | 7 |
| Growth factor receptor domain | 9 | 9 |
| GTPase activation domain, GAP | 9 | 7 |
| Histone H3 K4-specific methyltransferase SET7 or 9 N-terminal domain | 9 | 9 |
| HydB or Nqo4-like | 9 | 1 |
| MCP or YpsA-like | 9 | 9 |
| Release factor | 9 | 6 |
| Ricin B-like lectins | 9 | 10 |
| RNase III domain-like | 9 | 10 |
| SAP domain | 9 | 10 |
| SIS domain | 9 | 6 |
| TAZ domain | 9 | 5 |
| ThrRS or AlaRS common domain | 9 | 6 |
| TK C-terminal domain-like | 9 | 9 |
| TolB, C-terminal domain | 9 | 9 |
| AlbA-like | 8 | 6 |
| Alpha-L RNA-binding motif | 8 | 10 |
| Annexin | 8 | 8 |
| ARID-like | 8 | 8 |
| Cullin homology domain | 8 | 8 |
| DHS-like NAD or FAD-binding domain | 8 | 9 |
| Dihydrofolate reductase-like | 8 | 5 |
| FAD or NAD-linked reductases, dimerisation (C-terminal) domain | 8 | 7 |
| Flavoproteins | 8 | 8 |
| Histidine-containing phosphotransfer domain, HPT domain | 8 | 7 |
| HIT-like | 8 | 9 |
| HSP90 C-terminal domain | 8 | 7 |
| N-terminal domain of eukaryotic peptide chain release factor subunit 1, ERF1 | 8 | 4 |
| N-terminal, heterodimerisation domain of RBP7 (RpoE) | 8 | 8 |
| PLP-binding barrel | 8 | 8 |
| PYP-like sensor domain (PAS domain) | 8 | 8 |
| Restriction endonuclease-like | 8 | 8 |
| Ribosomal proteins L15p and L18e | 8 | 8 |
| Ribosomal proteins S24e, L23 and L15e | 8 | 10 |
| Rudiment single hybrid motif | 8 | 6 |
| Sec7 domain | 8 | 7 |
| Serpins | 8 | 9 |
| SMR domain-like | 8 | 8 |
| Surp module (SWAP domain) | 8 | 8 |
| TGS-like | 8 | 7 |
| Translation initiation factor 2 beta, aIF2beta, N-terminal domain | 8 | 7 |
| Tricorn protease domain 2 | 8 | 7 |
| Zinc-binding domain of translation initiation factor 2 beta | 8 | 7 |
| Acyl-CoA binding protein | 7 | 7 |
| Apolipoprotein A-I | 7 | 7 |
| ATP-dependent DNA ligase DNA-binding domain | 7 | 4 |
| Cation efflux protein cytoplasmic domain-like | 7 | 5 |
| Chalcone isomerase | 7 | 7 |
| CHY zinc finger-like | 7 | 6 |
| Clc chloride channel | 7 | 7 |
| ClpS-like | 7 | 7 |
| CSL zinc finger | 7 | 4 |
| Cysteine alpha-hairpin motif | 7 | 9 |
| EGF or Laminin | 7 | 9 |
| FAD-linked oxidases, C-terminal domain | 7 | 7 |
| Fe-S cluster assembly (FSCA) domain-like | 7 | 7 |
| Glutamine synthetase, N-terminal domain | 7 | 5 |
| Initiation factor IF2 or eIF5b, domain 3 | 7 | 4 |
| LDH C-terminal domain-like | 7 | 7 |
| Lipocalins | 7 | 8 |
| Macro domain-like | 7 | 5 |
| NHL repeat | 7 | 6 |
| Oxysterol-binding protein-like | 7 | 6 |
| PEBP-like | 7 | 8 |
| Peptidase or esterase 'gauge' domain | 7 | 6 |
| Phosphofructokinase | 7 | 6 |
| Protease propeptides or inhibitors | 7 | 6 |
| Ribosomal protein L1 | 7 | 7 |
| Ribosomal protein S8 | 7 | 6 |
| RuBisCo LSMT C-terminal, substrate-binding domain | 7 | 7 |
| RuvA domain 2-like | 7 | 6 |
| Sec1 or munc18-like (SM) proteins | 7 | 5 |
| Second domain of Mu2 adaptin subunit (ap50) of ap2 adaptor | 7 | 7 |
| SH3-domain | 7 | 6 |
| SpoIIaa-like | 7 | 7 |
| Zinc hairpin stack | 7 | 6 |
| Acyl-CoA dehydrogenase C-terminal domain-like | 6 | 6 |
| Acyl-CoA dehydrogenase NM domain-like | 6 | 6 |
| Alanine racemase C-terminal domain-like | 6 | 6 |
| Amine oxidase catalytic domain | 6 | 6 |
| Amine oxidase N-terminal region | 6 | 6 |
| Arginyl-tRNA synthetase (ArgRS), N-terminal 'additional' domain | 6 | 3 |
| BAG domain | 6 | 6 |
| Clathrin adaptor appendage domain | 6 | 4 |
| Composite domain of metallo-dependent hydrolases | 6 | 5 |
| Conserved domain common to transcription factors TFIIS, elongin A, CRSP70 | 6 | 7 |
| DEK C-terminal domain | 6 | 6 |
| DLC | 6 | 6 |
| DNA repair protein MutS, domain I | 6 | 4 |
| Domain of the SRP or SRP receptor G-proteins | 6 | 6 |
| Enolase C-terminal domain-like | 6 | 6 |
| Enolase N-terminal domain-like | 6 | 6 |
| ENT-like | 6 | 6 |
| Families 57 or 38 glycoside transferase middle domain | 6 | 6 |
| GINS helical bundle-like | 6 | 6 |
| GlnB-like | 6 | 5 |
| Glycoside hydrolase or deacetylase | 6 | 8 |
| Helical domain of Sec23 or 24 | 6 | 7 |
| JAB1 or MPN domain | 6 | 6 |
| L-aspartase-like | 6 | 6 |
| LeuD or IlvD-like | 6 | 5 |
| Magnesium transport protein CorA, transmembrane region | 6 | 6 |
| Mago nashi protein | 6 | 1 |
| Mitochondrial glycoprotein MAM33-like | 6 | 12 |
| MurD-like peptide ligases, catalytic domain | 6 | 6 |
| MurD-like peptide ligases, peptide-binding domain | 6 | 6 |
| Oxygen-evolving enhancer protein 3, | 6 | 5 |
| Peripheral subunit-binding domain of 2-oxo acid dehydrogenase complex | 6 | 5 |
| Poly A polymerase C-terminal region-like | 6 | 4 |
| Prokaryotic type KH domain (KH-domain type II) | 6 | 4 |
| PX domain | 6 | 6 |
| R3H domain | 6 | 5 |
| Ribosomal protein L14 | 6 | 3 |
| Ribosomal protein L29 (L29p) | 6 | 5 |
| Ribosomal protein L30p or L7e | 6 | 7 |
| Rubredoxin-like | 6 | 5 |
| Soluble quinoprotein glucose dehydrogenase | 6 | 5 |
| Supernatant protein factor (SPF), C-terminal domain | 6 | 7 |
| YVTN repeat-like or Quinoprotein amine dehydrogenase | 6 | 5 |
| Zn-finger domain of Sec23 or 24 | 6 | 7 |
| Actin-crosslinking proteins | 5 | 4 |
| ADC-like | 5 | 8 |
| AraD or HMP-PK domain-like | 5 | 1 |
| BEACH domain | 5 | 4 |
| C-terminal, gelsolin-like domain of Sec23 or 24 | 5 | 6 |
| Cdc48 domain 2-like | 5 | 8 |
| Chelatase | 5 | 6 |
| Cu,Zn superoxide dismutase-like | 5 | 5 |
| CYTH-like phosphatases | 5 | 9 |
| DNA repair protein MutS, domain II | 5 | 3 |
| DUSP-like | 5 | 4 |
| E2F-DP heterodimerization region | 5 | 5 |
| eIF1-like | 5 | 5 |
| F1F0 ATP synthase subunit C | 5 | 6 |
| HesB-like domain | 5 | 4 |
| Inorganic pyrophosphatase | 5 | 5 |
| KA1-like | 5 | 5 |
| L28p-like | 5 | 4 |
| L9 N-domain-like | 5 | 3 |
| Leukotriene A4 hydrolase N-terminal domain | 5 | 9 |
| Mss4-like | 5 | 6 |
| Nucleoside phosphorylase or phosphoribosyltransferase catalytic domain | 5 | 4 |
| Nucleoside phosphorylase or phosphoribosyltransferase N-terminal domain | 5 | 3 |
| Outer arm dynein light chain 1 | 5 | 6 |
| PABC (PABP) domain | 5 | 5 |
| Phosphoglucomutase, first 3 domains | 5 | 5 |
| Phosphoglycerate kinase | 5 | 4 |
| Preprotein translocase SecY subunit | 5 | 5 |
| PriA or YqbF domain | 5 | 5 |
| Profilin (actin-binding protein) | 5 | 3 |
| Putative isomerase YbhE | 5 | 5 |
| RBP11-like subunits of RNA polymerase | 5 | 4 |
| Riboflavin kinase-like | 5 | 5 |
| Ribosomal L27 protein-like | 5 | 5 |
| Ribosomal protein L6 | 5 | 5 |
| Ribosomal protein S3 C-terminal domain | 5 | 2 |
| Ribosomal protein S7 | 5 | 7 |
| Seven-hairpin glycosidases | 5 | 4 |
| Sigma2 domain of RNA polymerase sigma factors | 5 | 5 |
| Sigma3 and sigma4 domains of RNA polymerase sigma factors | 5 | 5 |
| ssDNA-binding transcriptional regulator domain | 5 | 6 |
| Tetrahydrobiopterin biosynthesis enzymes-like | 5 | 5 |
| Thymidylate synthase or dCMP hydroxymethylase | 5 | 2 |
| Tim10-like | 5 | 5 |
| UROD or MetE-like | 5 | 5 |
| YrdC or RibB | 5 | 4 |
| ADC synthase | 4 | 7 |
| alpha-helical ferredoxin | 4 | 4 |
| AMPKBI-like | 4 | 3 |
| BolA-like | 4 | 4 |
| C-terminal autoproteolytic domain of nucleoporin nup98 | 4 | 3 |
| C-terminal domain of adenylylcyclase associated protein | 4 | 4 |
| C-terminal domain of ProRS | 4 | 3 |
| Citrate synthase | 4 | 4 |
| Coiled-coil domain of nucleotide exchange factor GrpE | 4 | 4 |
| Creatinase or prolidase N-terminal domain | 4 | 3 |
| Cytochrome c | 4 | 4 |
| D-ribose-5-phosphate isomerase (RpiA), lid domain | 4 | 3 |
| DNA topoisomerase IV, alpha subunit | 4 | 4 |
| eEF-1beta-like | 4 | 2 |
| eIF4e-like | 4 | 4 |
| EPT or RTPC-like | 4 | 3 |
| Eukaryotic RPB5 N-terminal domain | 4 | 4 |
| Expressed protein At2g23090 or F21P24.15 | 4 | 4 |
| Glycolipid transfer protein, GLTP | 4 | 4 |
| Granulin repeat | 4 | 4 |
| Heme oxygenase-like | 4 | 4 |
| Inosine monophosphate dehydrogenase (IMPDH) | 4 | 7 |
| Lesion bypass DNA polymerase (Y-family), little finger domain | 4 | 3 |
| Methylglyoxal synthase-like | 4 | 3 |
| NAP-like | 4 | 4 |
| Nucleoside diphosphate kinase, NDK | 4 | 4 |
| Obg GTP-binding protein N-terminal domain | 4 | 4 |
| P-domain of calnexin or calreticulin | 4 | 3 |
| PAH2 domain | 4 | 5 |
| PAP or Archaeal CCA-adding enzyme, C-terminal domain | 4 | 4 |
| Pentapeptide repeat-like | 4 | 4 |
| Phospholipase C or P1 nuclease | 4 | 4 |
| PHP domain-like | 4 | 3 |
| Preprotein translocase SecE subunit | 4 | 2 |
| Probable bacterial effector-binding domain | 4 | 5 |
| Protein prenylyltransferase | 4 | 4 |
| Purine and uridine phosphorylases | 4 | 4 |
| Ribosomal L11 or L12e N-terminal domain | 4 | 4 |
| Ribosomal protein L10-like | 4 | 4 |
| Ribosomal protein L11, C-terminal domain | 4 | 4 |
| Ribosomal protein L13 | 4 | 4 |
| Ribosomal protein L22 | 4 | 3 |
| Ribosomal protein L4 | 4 | 3 |
| Ribosomal protein S10 | 4 | 5 |
| RL5-like | 4 | 4 |
| RPB5-like RNA polymerase subunit | 4 | 4 |
| S-adenosylmethionine decarboxylase | 4 | 5 |
| S15 or NS1 RNA-binding domain | 4 | 4 |
| Scorpion toxin-like | 4 | 24 |
| Stabilizer of iron transporter SufD | 4 | 2 |
| Staphylococcal nuclease | 4 | 3 |
| STI-like | 4 | 4 |
| Succinyl-CoA synthetase domains | 4 | 4 |
| SufE or NifU | 4 | 4 |
| Superfamily description | 4 | 4 |
| Tetrapyrrole methylase | 4 | 4 |
| The spindle assembly checkpoint protein mad2 | 4 | 4 |
| Toll or Interleukin receptor TIR domain | 4 | 4 |
| Transducin (alpha subunit), insertion domain | 4 | 4 |
| tRNA-binding arm | 4 | 5 |
| Type II DNA topoisomerase | 4 | 3 |
| UDP-glucose or GDP-mannose dehydrogenase C-terminal domain | 4 | 2 |
| Undecaprenyl diphosphate synthase | 4 | 6 |
| YWTD domain | 4 | 3 |
| 2,3-Bisphosphoglycerate-independent phosphoglycerate mutase, substrate-binding domain | 3 | 2 |
| 3-carboxy-cis,cis-mucoante lactonizing enzyme | 3 | 2 |
| 4'-phosphopantetheinyl transferase | 3 | 3 |
| Aconitase iron-sulfur domain | 3 | 3 |
| Alpha subunit of glutamate synthase, C-terminal domain | 3 | 3 |
| An anticodon-binding domain of class I aminoacyl-tRNA synthetases | 3 | 2 |
| ApaG-like | 3 | 2 |
| Arp2 or 3 complex subunits | 3 | 3 |
| Aspartate or ornithine carbamoyltransferase | 3 | 2 |
| ATP synthase (F1-ATPase), gamma subunit | 3 | 3 |
| B-box zinc-binding domain | 3 | 3 |
| beta-Galactosidase or glucuronidase domain | 3 | 3 |
| beta-N-acetylhexosaminidase-like domain | 3 | 3 |
| C-terminal domain of alpha and beta subunits of F1 ATP synthase | 3 | 5 |
| Caspase-like | 3 | 3 |
| CoaB-like | 3 | 1 |
| Cytochrome c oxidase subunit h | 3 | 4 |
| DNA clamp | 3 | 3 |
| Dom34 or Pelota N-terminal domain-like | 3 | 3 |
| eIF-2-alpha, C-terminal domain | 3 | 3 |
| eIF2alpha middle domain-like | 3 | 3 |
| Elongation factor TFIIS domain 2 | 3 | 3 |
| Endosomal sorting complex assembly domain | 3 | 4 |
| FAD-linked oxidoreductase | 3 | 3 |
| Fe,Mn superoxide dismutase (SOD), C-terminal domain | 3 | 3 |
| Fe,Mn superoxide dismutase (SOD), N-terminal domain | 3 | 3 |
| FF domain | 3 | 3 |
| Fibronectin type III | 3 | 3 |
| FMT C-terminal domain-like | 3 | 2 |
| Formyltransferase | 3 | 3 |
| Globin-like | 3 | 2 |
| Head domain of nucleotide exchange factor GrpE | 3 | 3 |
| Heme-dependent catalase-like | 3 | 3 |
| Homo-oligomeric flavin-containing Cys decarboxylases, HFCD | 3 | 3 |
| Homocysteine S-methyltransferase | 3 | 3 |
| Hypothetical protein YjiA, C-terminal domain | 3 | 3 |
| Indigoidine synthase A-like | 3 | 5 |
| Interferon-induced guanylate-binding protein 1 (GBP1), C-terminal domain | 3 | 3 |
| Isochorismatase-like hydrolases | 3 | 3 |
| lambda repressor-like DNA-binding domains | 3 | 3 |
| Ligand-binding domain in the NO signalling and Golgi transport | 3 | 3 |
| LigT-like | 3 | 3 |
| MAL13P1.257-like | 3 | 3 |
| N-terminal domain of alpha and beta subunits of F1 ATP synthase | 3 | 5 |
| N-terminal domain of the delta subunit of the F1F0-ATP synthase | 3 | 3 |
| Nitrite and sulphite reductase 4Fe-4S domain-like | 3 | 3 |
| Nucleoporin domain | 3 | 3 |
| Nucleoside hydrolase | 3 | 3 |
| Nucleotide-binding domain | 3 | 3 |
| Oxidoreductase molybdopterin-binding domain | 3 | 3 |
| PCD-like | 3 | 2 |
| Peptide deformylase | 3 | 3 |
| Peptide methionine sulfoxide reductase | 3 | 5 |
| Peptidyl-tRNA hydrolase II | 3 | 3 |
| Phosphoglucomutase, C-terminal domain | 3 | 3 |
| Phospholipase A2, PLA2 | 3 | 3 |
| Prokaryotic type I DNA topoisomerase | 3 | 3 |
| PUG domain-like | 3 | 2 |
| Putative anticodon-binding domain of alanyl-tRNA synthetase (AlaRS) | 3 | 2 |
| PWI domain | 3 | 2 |
| Rad51 N-terminal domain-like | 3 | 2 |
| Rap30 or 74 interaction domains | 3 | 3 |
| RibA-like | 3 | 2 |
| Ribosomal protein L16p or L10e | 3 | 3 |
| Ribosomal protein L19 (L19e) | 3 | 2 |
| Ribosomal protein L9 C-domain | 3 | 2 |
| Ribosomal protein S19 | 3 | 2 |
| Ribosomal protein S2 | 3 | 5 |
| RNA polymerase subunits | 3 | 3 |
| RuBisCO, small subunit | 3 | 3 |
| S13-like H2TH domain | 3 | 5 |
| Saposin | 3 | 3 |
| Signal peptide-binding domain | 3 | 3 |
| SPOC domain-like | 3 | 3 |
| Subdomain of clathrin and coatomer appendage domain | 3 | 3 |
| TATA-box binding protein-like | 3 | 6 |
| Transcription factor IIA (TFIIA), alpha-helical domain | 3 | 3 |
| Transcription factor IIA (TFIIA), beta-barrel domain | 3 | 3 |
| Transferrin receptor-like dimerisation domain | 3 | 3 |
| Translin | 3 | 2 |
| tRNA-guanine transglycosylase | 3 | 2 |
| VC0467-like | 3 | 3 |
| VHP, Villin headpiece domain | 3 | 3 |
| AbfB domain | 2 | 2 |
| Activator of Hsp90 ATPase, Aha1 | 2 | 2 |
| Aerolisin or ETX pore-forming domain | 2 | 2 |
| AF1104-like | 2 | 2 |
| Agglutinin | 2 | 2 |
| all-alpha NTP pyrophosphatases | 2 | 2 |
| Allene oxide cyclase-like | 2 | 2 |
| alpha-ketoacid dehydrogenase kinase, N-terminal domain | 2 | 2 |
| Aminomethyltransferase beta-barrel domain | 2 | 2 |
| Aminopeptidase or glucanase lid domain | 2 | 2 |
| Anticodon-binding domain of PheRS | 2 | 2 |
| Argininosuccinate synthetase, C-terminal domain | 2 | 2 |
| ATP synthase D chain-like | 2 | 1 |
| AtpF-like | 2 | 3 |
| beta-carbonic anhydrase, cab | 2 | 2 |
| C-terminal domain of Ku80 | 2 | 2 |
| C-terminal UvrC-binding domain of UvrB | 2 | 1 |
| Calpain large subunit, middle domain (domain III) | 2 | 1 |
| Carbamoyl phosphate synthetase, large subunit connection domain | 2 | 1 |
| Carbamoyl phosphate synthetase, small subunit N-terminal domain | 2 | 2 |
| Carboxypeptidase regulatory domain-like | 2 | 2 |
| Casein kinase II beta subunit | 2 | 2 |
| Chitinase insertion domain | 2 | 2 |
| Chorismate mutase II | 2 | 3 |
| CI-2 family of serine protease inhibitors | 2 | 7 |
| CO dehydrogenase flavoprotein C-terminal domain-like | 2 | 2 |
| CO dehydrogenase ISP C-domain like | 2 | 2 |
| CO dehydrogenase molybdoprotein N-domain-like | 2 | 2 |
| CofD-like | 2 | 1 |
| Cytochrome c1 subunit of cytochrome bc1 complex (Ubiquinol-cytochrome c reductase), transmembrane anchor | 2 | 2 |
| Diaminopimelate epimerase-like | 2 | 3 |
| DNA breaking-rejoining enzymes | 2 | 1 |
| DNA mismatch repair protein MutL | 2 | 2 |
| DNA-binding domain of EIN3-like | 2 | 2 |
| Domain of poly(ADP-ribose) polymerase | 2 | 4 |
| EB1 dimerisation domain-like | 2 | 2 |
| eEF1-gamma domain | 2 | 2 |
| Electron transport accessory proteins | 2 | 2 |
| Elongation factor Ts (EF-Ts), dimerisation domain | 2 | 2 |
| Epsilon subunit of mitochondrial F1F0-ATP synthase | 2 | 2 |
| ERO1-like | 2 | 1 |
| ERP29 C domain-like | 2 | 1 |
| Eukaryotic DNA topoisomerase I, N-terminal DNA-binding fragment | 2 | 1 |
| FAH | 2 | 2 |
| Fe-only hydrogenase | 2 | 2 |
| Folate-binding domain | 2 | 3 |
| Formiminotransferase domain of formiminotransferase-cyclodeaminase. | 2 | 2 |
| GAD domain-like | 2 | 2 |
| Gamma-glutamyl cyclotransferase-like | 2 | 3 |
| GIY-YIG endonuclease | 2 | 2 |
| Glutamyl tRNA-reductase catalytic, N-terminal domain | 2 | 2 |
| Glutamyl tRNA-reductase dimerization domain | 2 | 2 |
| GMP synthetase C-terminal dimerisation domain | 2 | 2 |
| Hairpin loop containing domain-like | 2 | 3 |
| HAND domain of the nucleosome remodeling ATPase ISWI | 2 | 2 |
| Helical scaffold and wing domains of SecA | 2 | 2 |
| Heme chaperone CcmE | 2 | 1 |
| Hypothetical protein c14orf129, hspc210 | 2 | 2 |
| Hypothetical protein SAV1430 | 2 | 2 |
| Hypothetical protein TM0160 | 2 | 3 |
| IlvD or EDD N-terminal domain-like | 2 | 1 |
| Insert subdomain of RNA polymerase alpha subunit | 2 | 2 |
| Integrin alpha N-terminal domain | 2 | 2 |
| ISP transmembrane anchor | 2 | 1 |
| ITPase-like | 2 | 2 |
| L21p-like | 2 | 2 |
| L35p-like | 2 | 2 |
| LCCL domain | 2 | 1 |
| LigB-like | 2 | 2 |
| Mannose 6-phosphate receptor domain | 2 | 2 |
| Mannose-binding lectins | 2 | 3 |
| MAPEG domain-like | 2 | 3 |
| Mediator hinge subcomplex-like | 2 | 2 |
| MIT domain | 2 | 2 |
| MoaD or ThiS | 2 | 2 |
| Molybdenum cofactor biosynthesis protein C, MoaC | 2 | 2 |
| Molybdenum cofactor-binding domain | 2 | 2 |
| Molybdopterin synthase subunit MoaE | 2 | 1 |
| MOSC N-terminal domain-like | 2 | 2 |
| MurCD N-terminal domain | 2 | 2 |
| MurE or MurF N-terminal domain | 2 | 2 |
| N-terminal domain of adenylylcyclase associated protein, CAP | 2 | 2 |
| N5-CAIR mutase (phosphoribosylaminoimidazole carboxylase, PurE) | 2 | 1 |
| NAD-binding domain of HMG-CoA reductase | 2 | 2 |
| Nitrite or Sulfite reductase N-terminal domain-like | 2 | 2 |
| Nqo1 FMN-binding domain-like | 2 | 1 |
| Nqo1 middle domain-like | 2 | 1 |
| Nqo1C-terminal domain-like | 2 | 1 |
| NusB-like | 2 | 2 |
| Pentein | 2 | 2 |
| Peptidyl-tRNA hydrolase-like | 2 | 4 |
| PFL-like glycyl radical enzymes | 2 | 2 |
| Photosystem II antenna protein-like | 2 | 1 |
| Pollen allergen ole e 6 | 2 | 2 |
| Polynucleotide phosphorylase or guanosine pentaphosphate synthase (PNPase or GPSI), domain 3 | 2 | 2 |
| PRC-barrel domain | 2 | 2 |
| Pre-protein crosslinking domain of SecA | 2 | 2 |
| Pre-PUA domain | 2 | 2 |
| Prim-pol domain | 2 | 2 |
| Prismane protein-like | 2 | 2 |
| Prokaryotic ribosomal protein L17 | 2 | 3 |
| PTPA-like | 2 | 2 |
| PurM C-terminal domain-like | 2 | 2 |
| PurM N-terminal domain-like | 2 | 2 |
| Putative cyclase | 2 | 3 |
| Putative DNA-binding domain | 2 | 2 |
| R1 subunit of ribonucleotide reductase, N-terminal domain | 2 | 2 |
| RbcX-like | 2 | 2 |
| RecA protein, C-terminal domain | 2 | 2 |
| Ribosomal protein L20 | 2 | 2 |
| Ribosomal protein L31e | 2 | 2 |
| Ribosomal protein S16 | 2 | 3 |
| Ribosomal protein S6 | 2 | 3 |
| Ribosome binding protein Y (YfiA homologue) | 2 | 2 |
| Ribosome recycling factor, RRF | 2 | 2 |
| RPB6 or omega subunit-like | 2 | 2 |
| RplX-like | 2 | 2 |
| Rps17e-like | 2 | 2 |
| S-adenosylmethionine synthetase | 2 | 4 |
| Sec-C motif | 2 | 2 |
| Serine metabolism enzymes domain | 2 | 1 |
| SH2 domain | 2 | 2 |
| Signal recognition particle alu RNA binding heterodimer, SRP9 or 14 | 2 | 2 |
| Substrate-binding domain of HMG-CoA reductase | 2 | 2 |
| Subunits of heterodimeric actin filament capping protein Capz | 2 | 2 |
| Succinate dehydrogenase or fumarate reductase flavoprotein C-terminal domain | 2 | 2 |
| Succinate dehydrogenase or fumarate reductase flavoprotein, catalytic domain | 2 | 2 |
| SurE-like | 2 | 2 |
| TAF(II)230 TBP-binding fragment | 2 | 2 |
| Tautomerase or MIF | 2 | 2 |
| THUMP domain-like | 2 | 2 |
| Translation initiation factor IF3, C-terminal domain | 2 | 2 |
| Translation initiation factor IF3, N-terminal domain | 2 | 2 |
| Trigger factor ribosome-binding domain | 2 | 2 |
| Triosephosphate isomerase (TIM) | 2 | 2 |
| tRNA-intron endonuclease catalytic domain-like | 2 | 3 |
| Tubulin chaperone cofactor A | 2 | 2 |
| UraD-Like | 2 | 2 |
| VPS9 domain | 2 | 1 |
| WGR domain-like | 2 | 4 |
| XPC-binding domain | 2 | 2 |
| YbaB-like | 2 | 1 |
| YbaK or ProRS associated domain | 2 | 2 |
| YgfY-like | 2 | 2 |
| YggU-like | 2 | 2 |
| (2r)-phospho-3-sulfolactate synthase ComA | 1 | 1 |
| 1-deoxy-D-xylulose-5-phosphate reductoisomerase, C-terminal domain | 1 | 1 |
| 14 kDa protein of cytochrome bc1 complex (Ubiquinol-cytochrome c reductase) | 1 | 1 |
| 2-isopropylmalate synthase LeuA, allosteric (dimerisation) domain | 1 | 1 |
| 6-hydroxymethyl-7,8-dihydropterin pyrophosphokinase, HPPK | 1 | 1 |
| Acetamidase or Formamidase-like | 1 | 1 |
| Acetoacetate decarboxylase-like | 1 | 1 |
| AF0625-like | 1 | 1 |
| AMMECR1-like | 1 | 1 |
| Arp2 or 3 complex 16 kDa subunit ARPC5 | 1 | 1 |
| Arp2 or 3 complex 21 kDa subunit ARPC3 | 1 | 1 |
| ASF1-like | 1 | 1 |
| Aspartate or glutamate racemase | 1 | 1 |
| ATP12-like | 1 | 1 |
| Bacterial photosystem II reaction centre, L and M subunits | 1 | 3 |
| Bactericidal permeability-increasing protein, BPI | 1 | 1 |
| BB1717-like | 1 | 1 |
| beta-lactamase or transpeptidase-like | 1 | 2 |
| Bowman-Birk inhibitor, BBI | 1 | 3 |
| BRCA2 helical domain | 1 | 1 |
| BRCA2 tower domain | 1 | 1 |
| C-terminal (heme d1) domain of cytochrome cd1-nitrite reductase | 1 | 1 |
| C-type lectin-like | 1 | 1 |
| Cap-Gly domain | 1 | 1 |
| CarD-like | 1 | 1 |
| CBD9-like | 1 | 1 |
| Cell cycle regulatory proteins | 1 | 1 |
| Cgl1923-like | 1 | 1 |
| Chorismate synthase, AroC | 1 | 1 |
| CinA-like | 1 | 1 |
| Clathrin heavy-chain terminal domain | 1 | 1 |
| Cna protein B-type domain | 1 | 1 |
| Coproporphyrinogen III oxidase | 1 | 1 |
| CorA soluble domain-like | 1 | 1 |
| Ctag or Cox11 | 1 | 1 |
| Cyanase C-terminal domain | 1 | 1 |
| Cytochrome c oxidase subunit I-like | 1 | 1 |
| DAK1 or DegV-like | 1 | 1 |
| Dehydroquinate synthase-like | 1 | 1 |
| DhaL-like | 1 | 1 |
| DHH phosphoesterases | 1 | 1 |
| Dihydropteroate synthetase-like | 1 | 1 |
| DmpA or ArgJ-like | 1 | 1 |
| DNA polymerase beta, N-terminal domain-like | 1 | 1 |
| DNA primase core | 1 | 1 |
| EndoU-like | 1 | 1 |
| Epsilon subunit of F1F0-ATP synthase N-terminal domain | 1 | 1 |
| EreA or ChaN-like | 1 | 1 |
| ERH-like | 1 | 1 |
| FAD-dependent thiol oxidase | 1 | 1 |
| Family A G protein-coupled receptor-like | 1 | 1 |
| FdhE-like | 1 | 1 |
| Ferredoxin thioredoxin reductase (FTR), catalytic beta chain | 1 | 1 |
| FGAM synthase PurL, linker domain | 1 | 1 |
| FKBP12-rapamycin-binding domain of FKBP-rapamycin-associated protein (FRAP) | 1 | 1 |
| FMN-dependent nitroreductase-like | 1 | 1 |
| Formate dehydrogenase or DMSO reductase, domains 1-3 | 1 | 1 |
| Frataxin or Nqo15-like | 1 | 1 |
| Fucose-specific lectin | 1 | 1 |
| Fumarate reductase respiratory complex transmembrane subunits | 1 | 1 |
| Fumarylacetoacetate hydrolase, FAH, N-terminal domain | 1 | 1 |
| FYSH domain | 1 | 1 |
| GatB or YqeY motif | 1 | 1 |
| Glu-tRNAGln amidotransferase C subunit | 1 | 1 |
| GUN4-like | 1 | 1 |
| GyrA or ParC C-terminal domain-like | 1 | 1 |
| Helical backbone metal receptor | 1 | 1 |
| HemD-like | 1 | 2 |
| HI0933 insert domain-like | 1 | 1 |
| HisI-like | 1 | 1 |
| Homing endonucleases | 1 | 1 |
| HSC20 (HSCB), C-terminal oligomerisation domain | 1 | 1 |
| HydA or Nqo6-like | 1 | 1 |
| Hypothetical protein AF0491, middle domain | 1 | 1 |
| Hypothetical protein At3g22680 | 1 | 1 |
| Hypothetical protein MG354 | 1 | 1 |
| Hypothetical protein PA1324 | 1 | 1 |
| Indolic compounds 2,3-dioxygenase-like | 1 | 1 |
| Invasin or intimin cell-adhesion fragments | 1 | 1 |
| IpsF-like | 1 | 1 |
| ISY1 domain-like | 1 | 1 |
| Kix domain of CBP (creb binding protein) | 1 | 3 |
| LanC-like | 1 | 1 |
| LmbE-like | 1 | 1 |
| Lumazine synthase | 1 | 1 |
| Malate synthase G | 1 | 1 |
| Mechanosensitive channel protein MscS (YggB), transmembrane region | 1 | 1 |
| Methionyl-tRNA synthetase (MetRS), Zn-domain | 1 | 1 |
| MHC antigen-recognition domain | 1 | 1 |
| MIR domain | 1 | 1 |
| Mitochondrial cytochrome c oxidase subunit VIa | 1 | 1 |
| Mitotic arrest deficient-like 1, Mad1 | 1 | 1 |
| Mob1 or phocein | 1 | 1 |
| MoeA C-terminal domain-like | 1 | 1 |
| MoeA N-terminal region -like | 1 | 1 |
| Molybdenum cofactor biosynthesis proteins | 1 | 1 |
| MTH938-like | 1 | 1 |
| Multidrug efflux transporter AcrB transmembrane domain | 1 | 1 |
| Multiheme cytochromes | 1 | 1 |
| Myosin rod fragments | 1 | 1 |
| N-utilization substance G protein NusG, N-terminal domain | 1 | 1 |
| NadA-like | 1 | 1 |
| Nicotinate or Quinolinate PRTase C-terminal domain-like | 1 | 2 |
| Nicotinate or Quinolinate PRTase N-terminal domain-like | 1 | 2 |
| NOB1 zinc finger-like | 1 | 1 |
| Non-heme 11 kDa protein of cytochrome bc1 complex (Ubiquinol-cytochrome c reductase) | 1 | 1 |
| Nop10-like SnoRNP | 1 | 1 |
| NSFL1 (p97 ATPase) cofactor p47, SEP domain | 1 | 1 |
| Nuclear receptor ligand-binding domain | 1 | 1 |
| Obg GTP-binding protein C-terminal domain | 1 | 1 |
| occludin or ELL-like | 1 | 1 |
| Oligoxyloglucan reducing end-specific cellobiohydrolase | 1 | 1 |
| OMPA-like | 1 | 1 |
| ParB or Sulfiredoxin | 1 | 1 |
| PEP carboxykinase N-terminal domain | 1 | 2 |
| PEP carboxykinase-like | 1 | 2 |
| Peptidyl-tRNA hydrolase domain-like | 1 | 1 |
| PetM subunit of the cytochrome b6f complex | 1 | 1 |
| PF0523-like | 1 | 1 |
| Phage fibre proteins | 1 | 1 |
| Photosystem I reaction center subunit X, PsaK | 1 | 1 |
| Photosystem I reaction center subunit XI, PsaL | 1 | 1 |
| Photosystem I subunit PsaD | 1 | 1 |
| Plant lectins or antimicrobial peptides | 1 | 5 |
| Porphobilinogen deaminase (hydroxymethylbilane synthase), C-terminal domain | 1 | 1 |
| Probable ACP-binding domain of malonyl-CoA ACP transacylase | 1 | 1 |
| PRP4-like | 1 | 2 |
| PsbU or PolX domain-like | 1 | 1 |
| PSPTO4464-like | 1 | 1 |
| PurS-like | 1 | 1 |
| Putative methyltransferase TM0872, insert domain | 1 | 1 |
| Pym (Within the bgcn gene intron protein, WIBG), N-terminal domain | 1 | 1 |
| Pyrrolidone carboxyl peptidase (pyroglutamate aminopeptidase) | 1 | 1 |
| Regulator of G-protein signaling, RGS | 1 | 1 |
| Ribosomal protein L39e | 1 | 1 |
| Ribosomal protein L7 or 12, oligomerisation (N-terminal) domain | 1 | 1 |
| Ribosomal protein S18 | 1 | 1 |
| Ribosomal protein S20 | 1 | 1 |
| Ribosome-binding factor A, RbfA | 1 | 1 |
| RNA polymerase subunit RPB10 | 1 | 1 |
| Rnp2-like | 1 | 2 |
| Rof or RNase P subunit-like | 1 | 1 |
| RraA-like | 1 | 1 |
| RuBisCo, C-terminal domain | 1 | 1 |
| SCP-like | 1 | 1 |
| Second domain of FERM | 1 | 1 |
| Sialidases | 1 | 1 |
| SMI1 or KNR4-like | 1 | 1 |
| ssDNA viruses | 1 | 1 |
| SSo0622-like | 1 | 1 |
| Subunit III of photosystem I reaction centre, PsaF | 1 | 1 |
| Subunit IX of photosystem I reaction centre, PsaJ | 1 | 1 |
| Subunit X (non-heme 7 kDa protein) of cytochrome bc1 complex (Ubiquinol-cytochrome c reductase) | 1 | 1 |
| Taf5 N-terminal domain-like | 1 | 1 |
| Tex N-terminal region-like | 1 | 1 |
| TFB5-like | 1 | 1 |
| Thiamin phosphate synthase | 1 | 1 |
| Thiamin pyrophosphokinase, catalytic domain | 1 | 1 |
| Thiamin pyrophosphokinase, substrate-binding domain | 1 | 1 |
| Transmembrane di-heme cytochromes | 1 | 1 |
| Transthyretin (synonym_alia.txt | 1 | 1 |
| TRAP-like | 1 | 1 |
| TRCF domain-like | 1 | 1 |
| Triger factor or SurA peptide-binding domain-like | 1 | 1 |
| TrkA C-terminal domain-like | 1 | 1 |
| Trm112p-like | 1 | 1 |
| TSP-1 type 1 repeat | 1 | 1 |
| TSP9-like | 1 | 1 |
| TTHA0068-like | 1 | 1 |
| Ubiquinone-binding protein QP-C of cytochrome bc1 complex (Ubiquinol-cytochrome c reductase) | 1 | 1 |
| Urease, beta-subunit | 1 | 1 |
| Urease, gamma-subunit | 1 | 1 |
| Uridine diphospho-N-Acetylenolpyruvylglucosamine reductase, MurB, C-terminal domain | 1 | 1 |
| V-type ATP synthase subunit C | 1 | 1 |
| V-type ATPase subunit E-like | 1 | 1 |
| VPS28 C-terminal domain-like | 1 | 1 |
| XRCC4, C-terminal oligomerization domain | 1 | 1 |
| XRCC4, N-terminal domain | 1 | 1 |
| Xylose isomerase-like | 1 | 1 |
| YaeB-like | 1 | 1 |
| YbeD or HP0495-like | 1 | 1 |
| YbiA-like | 1 | 1 |
| YccV-like | 1 | 1 |
| YebC-like | 1 | 1 |
| YgbK-like | 1 | 1 |
| YhbC-like, N-terminal domain | 1 | 1 |
| YjbQ-like | 1 | 1 |
| YjeF N-terminal domain-like | 1 | 1 |
| YjgF-like | 1 | 1 |
| Cytochrome c oxidase subunit III-like | 0 | 1 |
| Cytochrome f subunit of the cytochrome b6f complex, transmembrane anchor | 0 | 1 |
| Cytochrome f, large domain | 0 | 1 |
| Dcp2 domain-like | 0 | 1 |
| DTD-like | 0 | 1 |
| dUTPase-like | 0 | 4 |
| F1F0 ATP synthase subunit A | 0 | 1 |
| Functional domain of the splicing factor Prp18 | 0 | 1 |
| Hermes dimerisation domain | 0 | 1 |
| Nitrous oxide reductase, N-terminal domain | 0 | 1 |
| Nqo5-like | 0 | 1 |
| Oligosaccharyltransferase subunit ost4p | 0 | 1 |
| PetG subunit of the cytochrome b6f complex | 0 | 2 |
| PetN subunit of the cytochrome b6f complex | 0 | 1 |
| Phosphotyrosine protein phosphatases I | 0 | 1 |
| Photosystem I subunits PsaA or PsaB | 0 | 1 |
| Photosystem II 10 kDa phosphoprotein PsbH | 0 | 1 |
| Photosystem II reaction center protein I, PsbI | 0 | 1 |
| Photosystem II reaction center protein J, PsbJ | 0 | 1 |
| Photosystem II reaction center protein K, PsbK | 0 | 1 |
| Photosystem II reaction center protein L, PsbL | 0 | 1 |
| Photosystem II reaction center protein T, PsbT | 0 | 1 |
| Rho N-terminal domain-like | 0 | 1 |
| Ribose or Galactose isomerase RpiB or AlsB | 0 | 5 |
| Ribosomal protein L32e | 0 | 2 |
| Ribosomal protein L36 | 0 | 2 |
| SRP19 | 0 | 2 |
| Subunit VIII of photosystem I reaction centre, PsaI | 0 | 1 |
| Uracil-DNA glycosylase-like | 0 | 4 |
| Vacuolar ATP synthase subunit C | 0 | 2 |

**Table S10.** Significance tests of gene density between A.ox_chr4 and other chromosomes.

|  | **Comparison between A.ox_chr4 and other chromosomes** | | | | | |
| --- | --- | --- | --- | --- | --- | --- |
|  | A.ox_chr1 | A.ox_chr2 | A.ox_chr3 | A.ox_chr5 | A.ox_chr6 | A.ox_chr7 |
| *P*-adj | 0.01 | 2.4×10^-3^ | 1.9×10^-5^ | 1.8×10^-5^ | 1.8×10^-5^ | 6.1×10^-4^ |

**Table S11.** Significance tests of *d*_N_/*d*_S_ values between one-to-one orthologs on A.ox_chr4 and other chromosomes.

|  | **Comparison between A.ox_chr4 and other chromosomes** | | | | | |
| --- | --- | --- | --- | --- | --- | --- |
|  | A.ox_chr1 | A.ox_chr2 | A.ox_chr3 | A.ox_chr5 | A.ox_chr6 | A.ox_chr7 |
| *P*-adj | 0.3 | 5.7×10^-3^ | 0.03 | 0.05 | 0.11 | 6.4×10^-4^ |

**Table S12.** Significance tests of gene density between A.co_chr4-homologous segments and other chromosomes or chromosome segments.

|  | ***P*-adj from diagonal comparison** | | | | | | | | |
| --- | --- | --- | --- | --- | --- | --- | --- | --- | --- |
|  | A.ox_chr1_1 | A.ox_chr1_2 | A.ox_chr2 | A.ox_chr3 | A.ox_chr4_1 | A.ox_chr4_2 | A.ox_chr5 | A.ox_chr6 | A.ox_chr7 |
| A.ox_chr1_2 | 1.4×10^-4^ | - | 6.8×10^-4^ | 2.1×10^-5^ | 0.2 | 4.8×10^-10^ | 2.1×10^-5^ | 2.1×10^-5^ | 3.0×10^-4^ |
| A.ox_chr4_1 | 9.9×10^-9^ | 0.2 | 6.5×10^-8^ | 1.9×10^-11^ | - | 3.0×10^-13^ | 1.9×10^-11^ | 5.6×10^-11^ | 1.5×10^-8^ |

**Table S13.** Significance tests of *d*_N_/*d*_S_ values between one-to-one orthologs on the two A.co_chr4-homologous chromosome segments and those on other chromosomes or other chromosome segments.

|  | ***P*-adj from diagonal comparison** | | | | | | | | |
| --- | --- | --- | --- | --- | --- | --- | --- | --- | --- |
|  | A.ox_chr1_1 | A.ox_chr1_2 | A.ox_chr2 | A.ox_chr3 | A.ox_chr4_1 | A.ox_chr4_2 | A.ox_chr5 | A.ox_chr6 | A.ox_chr7 |
| A.ox_chr1_2 | 0.16 | - | 0.04 | 0.09 | 0.30 | 0.16 | 0.10 | 0.13 | 0.02 |
| A.ox_chr4_1 | 0.03 | 0.30 | 1.2×10^-3^ | 4.6×10^-3^ | - | 0.03 | 7.1×10^-3^ | 0.02 | 2.8×10^-4^ |

**Table S14.** Genome coordinates of 5S rDNA loci in *A*. *oxysepala* var. *kansuensis*.

| **ID^1^** | **Chromosome** | **Start** | **End** |
| --- | --- | --- | --- |
| 5S_rRNA_0001 | chr1 | 33923773 | 33923894 |
| 5S_rRNA_0002 | chr1 | 33924506 | 33924627 |
| 5S_rRNA_0003 | chr1 | 33925604 | 33925725 |
| 5S_rRNA_0004 | chr1 | 33926335 | 33926456 |
| 5S_rRNA_0005 | chr1 | 33926702 | 33926823 |
| 5S_rRNA_0006 | chr1 | 33927068 | 33927189 |
| 5S_rRNA_0007 | chr1 | 33927434 | 33927555 |
| 5S_rRNA_0008 | chr1 | 33927800 | 33927921 |
| 5S_rRNA_0009 | chr1 | 33928164 | 33928285 |
| 5S_rRNA_0010 | chr1 | 33928530 | 33928651 |
| 5S_rRNA_0011 | chr1 | 33928896 | 33929017 |
| 5S_rRNA_0012 | chr1 | 33929262 | 33929383 |
| 5S_rRNA_0013 | chr1 | 33924140 | 33924259 |
| 5S_rRNA_0014 | chr1 | 33924875 | 33924994 |
| 5S_rRNA_0015 | chr1 | 33925240 | 33925359 |
| 5S_rRNA_0016 | chr1 | 33925971 | 33926090 |
| 5S_rRNA_0017 | chr1 | 33929630 | 33929749 |
| 5S_rRNA_0018 | chr1 | 33929997 | 33930115 |
| 5S_rRNA_0019 | chr1 | 33923416 | 33923528 |
| 5S_rRNA_0020 | chr1 | 34790365 | 34790444 |
| 5S_rRNA_0021 | chr1 | 38996929 | 38997042 |
| 5S_rRNA_0022 | chr1 | 15215141 | 15215260 |
| 5S_rRNA_0023 | chr1 | 38998583 | 38998697 |
| 5S_rRNA_0024 | chr1 | 38997277 | 38997396 |
| 5S_rRNA_0025 | chr1 | 38997602 | 38997719 |
| 5S_rRNA_0026 | chr1 | 38998964 | 38999046 |
| 5S_rRNA_0027 | chr1 | 38999297 | 38999368 |
| 5S_rRNA_0028 | chr1 | 2542608 | 2542670 |
| 5S_rRNA_0029 | chr1 | 38205327 | 38205373 |
| 5S_rRNA_0030 | chr1 | 42959141 | 42959203 |
| 5S_rRNA_0031 | chr1 | 33930381 | 33930430 |
| 5S_rRNA_0032 | chr1 | 42741624 | 42741680 |
| 5S_rRNA_0085 | chr2 | 26831746 | 26831810 |
| 5S_rRNA_0086 | chr2 | 40990409 | 40990525 |
| 5S_rRNA_0113 | chr3 | 27093777 | 27093839 |
| 5S_rRNA_0114 | chr3 | 32623667 | 32623713 |
| 5S_rRNA_0115 | chr3 | 27322398 | 27322460 |
| 5S_rRNA_0133 | chr4 | 26832878 | 26832999 |
| 5S_rRNA_0134 | chr4 | 26833244 | 26833365 |
| 5S_rRNA_0135 | chr4 | 26833610 | 26833731 |
| 5S_rRNA_0136 | chr4 | 26833975 | 26834096 |
| 5S_rRNA_0137 | chr4 | 26834341 | 26834462 |
| 5S_rRNA_0138 | chr4 | 26834707 | 26834828 |
| 5S_rRNA_0139 | chr4 | 26835065 | 26835186 |
| 5S_rRNA_0140 | chr4 | 26835430 | 26835551 |
| 5S_rRNA_0141 | chr4 | 26835796 | 26835917 |
| 5S_rRNA_0142 | chr4 | 26836153 | 26836274 |
| 5S_rRNA_0143 | chr4 | 26836518 | 26836639 |
| 5S_rRNA_0144 | chr4 | 26836884 | 26837005 |
| 5S_rRNA_0145 | chr4 | 26837250 | 26837371 |
| 5S_rRNA_0146 | chr4 | 26837607 | 26837728 |
| 5S_rRNA_0147 | chr4 | 26837964 | 26838085 |
| 5S_rRNA_0148 | chr4 | 26838321 | 26838442 |
| 5S_rRNA_0149 | chr4 | 26838687 | 26838808 |
| 5S_rRNA_0150 | chr4 | 26839053 | 26839174 |
| 5S_rRNA_0151 | chr4 | 26839419 | 26839540 |
| 5S_rRNA_0152 | chr4 | 26839785 | 26839906 |
| 5S_rRNA_0153 | chr4 | 26840150 | 26840271 |
| 5S_rRNA_0154 | chr4 | 26840514 | 26840635 |
| 5S_rRNA_0155 | chr4 | 26840880 | 26841001 |
| 5S_rRNA_0156 | chr4 | 26841237 | 26841358 |
| 5S_rRNA_0157 | chr4 | 26841602 | 26841723 |
| 5S_rRNA_0158 | chr4 | 26841959 | 26842080 |
| 5S_rRNA_0159 | chr4 | 26842325 | 26842446 |
| 5S_rRNA_0160 | chr4 | 26842682 | 26842803 |
| 5S_rRNA_0161 | chr4 | 26843039 | 26843160 |
| 5S_rRNA_0162 | chr4 | 26843396 | 26843517 |
| 5S_rRNA_0163 | chr4 | 26843762 | 26843883 |
| 5S_rRNA_0164 | chr4 | 26844129 | 26844250 |
| 5S_rRNA_0165 | chr4 | 26844494 | 26844615 |
| 5S_rRNA_0166 | chr4 | 26844860 | 26844981 |
| 5S_rRNA_0167 | chr4 | 26845226 | 26845347 |
| 5S_rRNA_0168 | chr4 | 26845583 | 26845704 |
| 5S_rRNA_0169 | chr4 | 26845948 | 26846069 |
| 5S_rRNA_0170 | chr4 | 26848322 | 26848443 |
| 5S_rRNA_0171 | chr4 | 26848688 | 26848809 |
| 5S_rRNA_0172 | chr4 | 26849054 | 26849175 |
| 5S_rRNA_0173 | chr4 | 26849420 | 26849541 |
| 5S_rRNA_0174 | chr4 | 26849787 | 26849908 |
| 5S_rRNA_0175 | chr4 | 26850153 | 26850274 |
| 5S_rRNA_0176 | chr4 | 26850519 | 26850640 |
| 5S_rRNA_0177 | chr4 | 26850885 | 26851006 |
| 5S_rRNA_0178 | chr4 | 26851252 | 26851373 |
| 5S_rRNA_0179 | chr4 | 26851618 | 26851739 |
| 5S_rRNA_0180 | chr4 | 26851985 | 26852106 |
| 5S_rRNA_0181 | chr4 | 26852350 | 26852471 |
| 5S_rRNA_0182 | chr4 | 26852716 | 26852837 |
| 5S_rRNA_0183 | chr4 | 26853095 | 26853216 |
| 5S_rRNA_0184 | chr4 | 26853461 | 26853582 |
| 5S_rRNA_0185 | chr4 | 26853826 | 26853947 |
| 5S_rRNA_0186 | chr4 | 26854193 | 26854314 |
| 5S_rRNA_0187 | chr4 | 26854559 | 26854680 |
| 5S_rRNA_0188 | chr4 | 26854925 | 26855046 |
| 5S_rRNA_0189 | chr4 | 26855291 | 26855412 |
| 5S_rRNA_0190 | chr4 | 26855658 | 26855779 |
| 5S_rRNA_0191 | chr4 | 26856024 | 26856145 |
| 5S_rRNA_0192 | chr4 | 26856390 | 26856511 |
| 5S_rRNA_0193 | chr4 | 26856756 | 26856877 |
| 5S_rRNA_0194 | chr4 | 26857122 | 26857243 |
| 5S_rRNA_0195 | chr4 | 26857488 | 26857609 |
| 5S_rRNA_0196 | chr4 | 26857854 | 26857975 |
| 5S_rRNA_0197 | chr4 | 26858220 | 26858341 |
| 5S_rRNA_0198 | chr4 | 26858586 | 26858707 |
| 5S_rRNA_0199 | chr4 | 26858952 | 26859073 |
| 5S_rRNA_0200 | chr4 | 26868423 | 26868544 |
| 5S_rRNA_0201 | chr4 | 26868787 | 26868908 |
| 5S_rRNA_0202 | chr4 | 26869153 | 26869274 |
| 5S_rRNA_0203 | chr4 | 26869520 | 26869641 |
| 5S_rRNA_0204 | chr4 | 26869888 | 26870009 |
| 5S_rRNA_0205 | chr4 | 26870255 | 26870376 |
| 5S_rRNA_0206 | chr4 | 26870621 | 26870742 |
| 5S_rRNA_0207 | chr4 | 26870986 | 26871107 |
| 5S_rRNA_0208 | chr4 | 26876567 | 26876688 |
| 5S_rRNA_0209 | chr4 | 26879463 | 26879584 |
| 5S_rRNA_0210 | chr4 | 26880193 | 26880314 |
| 5S_rRNA_0211 | chr4 | 26886034 | 26886155 |
| 5S_rRNA_0212 | chr4 | 27620665 | 27620786 |
| 5S_rRNA_0213 | chr4 | 27624719 | 27624840 |
| 5S_rRNA_0214 | chr4 | 27626562 | 27626683 |
| 5S_rRNA_0215 | chr4 | 27627667 | 27627788 |
| 5S_rRNA_0216 | chr4 | 27628035 | 27628156 |
| 5S_rRNA_0217 | chr4 | 27631724 | 27631845 |
| 5S_rRNA_0218 | chr4 | 27633569 | 27633690 |
| 5S_rRNA_0219 | chr4 | 26880924 | 26881043 |
| 5S_rRNA_0220 | chr4 | 26881289 | 26881408 |
| 5S_rRNA_0221 | chr4 | 26883847 | 26883966 |
| 5S_rRNA_0222 | chr4 | 27625457 | 27625576 |
| 5S_rRNA_0223 | chr4 | 26868057 | 26868178 |
| 5S_rRNA_0224 | chr4 | 26882021 | 26882142 |
| 5S_rRNA_0225 | chr4 | 26882386 | 26882507 |
| 5S_rRNA_0226 | chr4 | 26882751 | 26882872 |
| 5S_rRNA_0227 | chr4 | 26884579 | 26884696 |
| 5S_rRNA_0228 | chr4 | 26884940 | 26885061 |
| 5S_rRNA_0229 | chr4 | 26885669 | 26885790 |
| 5S_rRNA_0230 | chr4 | 27619929 | 27620050 |
| 5S_rRNA_0231 | chr4 | 27622141 | 27622262 |
| 5S_rRNA_0232 | chr4 | 27623983 | 27624104 |
| 5S_rRNA_0233 | chr4 | 27624352 | 27624473 |
| 5S_rRNA_0234 | chr4 | 27626930 | 27627051 |
| 5S_rRNA_0235 | chr4 | 27628404 | 27628525 |
| 5S_rRNA_0236 | chr4 | 27629511 | 27629632 |
| 5S_rRNA_0237 | chr4 | 27630617 | 27630738 |
| 5S_rRNA_0238 | chr4 | 27632093 | 27632214 |
| 5S_rRNA_0239 | chr4 | 27633201 | 27633322 |
| 5S_rRNA_0240 | chr4 | 26879830 | 26879949 |
| 5S_rRNA_0241 | chr4 | 26883117 | 26883236 |
| 5S_rRNA_0242 | chr4 | 27622506 | 27622628 |
| 5S_rRNA_0243 | chr4 | 27625088 | 27625210 |
| 5S_rRNA_0244 | chr4 | 26847959 | 26848079 |
| 5S_rRNA_0245 | chr4 | 26871717 | 26871837 |
| 5S_rRNA_0246 | chr4 | 26877285 | 26877405 |
| 5S_rRNA_0247 | chr4 | 26877643 | 26877763 |
| 5S_rRNA_0248 | chr4 | 26879098 | 26879219 |
| 5S_rRNA_0249 | chr4 | 27620297 | 27620418 |
| 5S_rRNA_0250 | chr4 | 27621034 | 27621155 |
| 5S_rRNA_0251 | chr4 | 27621772 | 27621893 |
| 5S_rRNA_0252 | chr4 | 27622874 | 27622995 |
| 5S_rRNA_0253 | chr4 | 27625825 | 27625946 |
| 5S_rRNA_0254 | chr4 | 27630248 | 27630369 |
| 5S_rRNA_0255 | chr4 | 27630986 | 27631107 |
| 5S_rRNA_0256 | chr4 | 27632463 | 27632584 |
| 5S_rRNA_0257 | chr4 | 26873548 | 26873667 |
| 5S_rRNA_0258 | chr4 | 26878368 | 26878485 |
| 5S_rRNA_0259 | chr4 | 27623612 | 27623734 |
| 5S_rRNA_0260 | chr4 | 26871358 | 26871474 |
| 5S_rRNA_0261 | chr4 | 26880558 | 26880679 |
| 5S_rRNA_0262 | chr4 | 26883480 | 26883601 |
| 5S_rRNA_0263 | chr4 | 26885309 | 26885425 |
| 5S_rRNA_0264 | chr4 | 27621403 | 27621524 |
| 5S_rRNA_0265 | chr4 | 27623241 | 27623361 |
| 5S_rRNA_0266 | chr4 | 27626193 | 27626314 |
| 5S_rRNA_0267 | chr4 | 27629142 | 27629263 |
| 5S_rRNA_0268 | chr4 | 27629879 | 27630000 |
| 5S_rRNA_0269 | chr4 | 27633938 | 27634059 |
| 5S_rRNA_0270 | chr4 | 26878733 | 26878852 |
| 5S_rRNA_0271 | chr4 | 27631355 | 27631474 |
| 5S_rRNA_0272 | chr4 | 26873188 | 26873309 |
| 5S_rRNA_0273 | chr4 | 26881652 | 26881777 |
| 5S_rRNA_0274 | chr4 | 27627297 | 27627416 |
| 5S_rRNA_0275 | chr4 | 27628773 | 27628892 |
| 5S_rRNA_0276 | chr4 | 27632832 | 27632951 |
| 5S_rRNA_0277 | chr4 | 26872823 | 26872942 |
| 5S_rRNA_0278 | chr4 | 26878003 | 26878123 |
| 5S_rRNA_0279 | chr4 | 26884210 | 26884331 |
| 5S_rRNA_0280 | chr4 | 26876925 | 26877046 |
| 5S_rRNA_0281 | chr4 | 26872452 | 26872577 |
| 5S_rRNA_0282 | chr4 | 26872083 | 26872207 |
| 5S_rRNA_0283 | chr4 | 26873915 | 26874046 |
| 5S_rRNA_0284 | chr4 | 26874291 | 26874391 |
| 5S_rRNA_0285 | chr4 | 14382333 | 14382397 |
| 5S_rRNA_0286 | chr4 | 12465938 | 12465994 |
| 5S_rRNA_0287 | chr4 | 26847648 | 26847710 |
| 5S_rRNA_0288 | chr4 | 16900437 | 16900499 |
| 5S_rRNA_0289 | chr4 | 27619674 | 27619712 |
| 5S_rRNA_0290 | chr4 | 33260556 | 33260618 |
| 5S_rRNA_0302 | chr5 | 5871532 | 5871647 |
| 5S_rRNA_0303 | chr5 | 11055248 | 11055295 |
| 5S_rRNA_0304 | chr5 | 35056967 | 35057029 |
| 5S_rRNA_0305 | chr5 | 27303391 | 27303454 |
| 5S_rRNA_0306 | chr5 | 35159792 | 35159854 |
| 5S_rRNA_0319 | chr6 | 15604845 | 15604907 |
| 5S_rRNA_0321 | chr7 | 15812964 | 15813026 |
| 5S_rRNA_0383 | SUPER_188 | 381 | 502 |
| 5S_rRNA_0384 | SUPER_188 | 748 | 869 |
| 5S_rRNA_0385 | SUPER_188 | 1114 | 1235 |
| 5S_rRNA_0386 | SUPER_188 | 1480 | 1601 |
| 5S_rRNA_0387 | SUPER_188 | 1847 | 1968 |
| 5S_rRNA_0388 | SUPER_188 | 2213 | 2334 |
| 5S_rRNA_0389 | SUPER_188 | 2579 | 2700 |
| 5S_rRNA_0390 | SUPER_188 | 2945 | 3066 |
| 5S_rRNA_0391 | SUPER_188 | 3312 | 3433 |
| 5S_rRNA_0392 | SUPER_188 | 3678 | 3799 |
| 5S_rRNA_0393 | SUPER_188 | 4045 | 4166 |
| 5S_rRNA_0394 | SUPER_188 | 4412 | 4533 |
| 5S_rRNA_0395 | SUPER_188 | 4777 | 4898 |
| 5S_rRNA_0396 | SUPER_188 | 5142 | 5263 |
| 5S_rRNA_0397 | SUPER_188 | 5509 | 5630 |
| 5S_rRNA_0398 | SUPER_188 | 5875 | 5996 |
| 5S_rRNA_0399 | SUPER_188 | 6241 | 6362 |
| 5S_rRNA_0400 | SUPER_188 | 6607 | 6728 |
| 5S_rRNA_0401 | SUPER_188 | 6973 | 7094 |
| 5S_rRNA_0402 | SUPER_188 | 7338 | 7459 |
| 5S_rRNA_0403 | SUPER_188 | 8567 | 8688 |
| 5S_rRNA_0404 | SUPER_188 | 8933 | 9054 |
| 5S_rRNA_0405 | SUPER_188 | 9299 | 9420 |
| 5S_rRNA_0406 | SUPER_188 | 9664 | 9785 |
| 5S_rRNA_0407 | SUPER_188 | 10030 | 10151 |
| 5S_rRNA_0408 | SUPER_188 | 10397 | 10518 |
| 5S_rRNA_0409 | SUPER_188 | 10764 | 10885 |
| 5S_rRNA_0410 | SUPER_188 | 11129 | 11250 |
| 5S_rRNA_0411 | SUPER_188 | 11496 | 11617 |
| 5S_rRNA_0412 | SUPER_188 | 11862 | 11983 |
| 5S_rRNA_0413 | SUPER_188 | 12228 | 12349 |
| 5S_rRNA_0414 | SUPER_188 | 12594 | 12715 |
| 5S_rRNA_0415 | SUPER_188 | 12961 | 13082 |
| 5S_rRNA_0416 | SUPER_188 | 13328 | 13449 |
| 5S_rRNA_0417 | SUPER_188 | 13693 | 13814 |
| 5S_rRNA_0418 | SUPER_188 | 14059 | 14180 |
| 5S_rRNA_0419 | SUPER_188 | 14425 | 14546 |
| 5S_rRNA_0420 | SUPER_188 | 14791 | 14912 |
| 5S_rRNA_0421 | SUPER_188 | 15157 | 15278 |
| 5S_rRNA_0422 | SUPER_188 | 15523 | 15644 |
| 5S_rRNA_0423 | SUPER_188 | 15890 | 16011 |
| 5S_rRNA_0424 | SUPER_188 | 16257 | 16378 |
| 5S_rRNA_0425 | SUPER_188 | 16623 | 16744 |
| 5S_rRNA_0426 | SUPER_188 | 16989 | 17110 |
| 5S_rRNA_0427 | SUPER_188 | 17356 | 17477 |
| 5S_rRNA_0428 | SUPER_188 | 17723 | 17844 |
| 5S_rRNA_0429 | SUPER_188 | 18089 | 18210 |
| 5S_rRNA_0430 | SUPER_188 | 18455 | 18576 |
| 5S_rRNA_0431 | SUPER_188 | 19192 | 19313 |
| 5S_rRNA_0432 | SUPER_188 | 19558 | 19679 |
| 5S_rRNA_0433 | SUPER_188 | 19924 | 20045 |
| 5S_rRNA_0434 | SUPER_188 | 20290 | 20411 |
| 5S_rRNA_0435 | SUPER_188 | 20656 | 20777 |
| 5S_rRNA_0436 | SUPER_188 | 21022 | 21143 |
| 5S_rRNA_0437 | SUPER_188 | 21388 | 21509 |
| 5S_rRNA_0438 | SUPER_188 | 21754 | 21875 |
| 5S_rRNA_0439 | SUPER_188 | 22120 | 22241 |
| 5S_rRNA_0440 | SUPER_188 | 22486 | 22607 |
| 5S_rRNA_0441 | SUPER_188 | 22852 | 22973 |
| 5S_rRNA_0442 | SUPER_188 | 23218 | 23339 |
| 5S_rRNA_0443 | SUPER_188 | 23584 | 23705 |
| 5S_rRNA_0444 | SUPER_188 | 23950 | 24071 |
| 5S_rRNA_0445 | SUPER_188 | 24316 | 24437 |
| 5S_rRNA_0446 | SUPER_188 | 24682 | 24803 |
| 5S_rRNA_0447 | SUPER_188 | 25048 | 25169 |
| 5S_rRNA_0448 | SUPER_188 | 25414 | 25535 |
| 5S_rRNA_0449 | SUPER_188 | 25780 | 25901 |
| 5S_rRNA_0450 | SUPER_188 | 26146 | 26267 |
| 5S_rRNA_0451 | SUPER_188 | 26511 | 26632 |
| 5S_rRNA_0452 | SUPER_188 | 26878 | 26999 |
| 5S_rRNA_0453 | SUPER_188 | 27244 | 27365 |
| 5S_rRNA_0454 | SUPER_188 | 27610 | 27731 |
| 5S_rRNA_0455 | SUPER_188 | 27976 | 28097 |
| 5S_rRNA_0456 | SUPER_188 | 28342 | 28463 |
| 5S_rRNA_0457 | SUPER_188 | 28708 | 28829 |
| 5S_rRNA_0458 | SUPER_188 | 29073 | 29194 |
| 5S_rRNA_0459 | SUPER_188 | 29440 | 29561 |
| 5S_rRNA_0460 | SUPER_188 | 29805 | 29926 |
| 5S_rRNA_0461 | SUPER_188 | 30171 | 30292 |
| 5S_rRNA_0462 | SUPER_188 | 15 | 137 |
| 5S_rRNA_0463 | SUPER_188 | 7703 | 7817 |
| 5S_rRNA_0464 | SUPER_188 | 18821 | 18947 |
| 5S_rRNA_0465 | SUPER_477 | 366 | 429 |

^1^Note: for simplicity, the ID of each 5S rDNA gene here is abbreviated, for example, from “genome_rRNA_5S_0001” in the gff file for rRNA genes to “5S_rRNA_0001”.
